# Design and assessment of an intervention for parents of premature newborns: a mixed-methods study*

**DOI:** 10.1590/1518-8345.7791.4747

**Published:** 2026-02-02

**Authors:** Sandra Patricia Osorio-Galeano, Angela María Salazar-Maya

**Affiliations:** 1 Universidad de Antioquia, Facultad de Enfermería, Medellín, Antioquia, Colombia; 2 Universidad CES, Facultad de Enfermería, Medellín, Antioquia, Colombia

**Keywords:** Neonatal Nursing, Premature Infant, Parents, Neonatal Intensive Care Units, Empowerment, Nursing Research

## Abstract

to design and assess an intervention targeted at reinforcing parents’ competency in caring for premature newborns.

a mixed-methods, exploratory and sequential intervention study. A grounded theory was applied in the first phase. The participants were 12 mothers and 4 fathers. A randomized pilot study was implemented in the second phase, with 14 mothers in each group. The main response variable was “care competency”. The CUIDAR Ma-Pre scale was applied in the baseline and follow-up measurements.

four categories emerged: Facing premature births, Experiencing the prematurity routine in Neonatal Units, Self-empowerment to care for premature newborns, and Caring for premature newborns at home. Self-empowerment to care for premature newborns was the central category. These results were integrated into the intervention design. Differences in favor of the intervention were found in the assessment. Although these differences were not statistically significant between the groups, it were in fact so intra-group (Control n=13 baseline 82.15; at discharge 117.08 p=0.000 vs Intervention n=14 baseline 80.71; at discharge 121.14 p=0.000).

empowerment was essential in the experience; an intervention with this approach is relevant, and its preliminary assessment suggests that it can improve care competency. It is pertinent to conduct a study at a larger scale. ClinicalTrials.gov. Identifier: NCT05005988.

## Highlights

**(1)** Empowerment is central in the experience of caring for premature newborns. **(2)** It is pertinent to design an intervention based on the parents’ perspective. **(3)** The empowerment theoretical approach can be applied to educational interventions. **(4)** It is both viable and acceptable to devise an intervention targeted at parents of premature newborns. **(5)** Empowerment might reinforce care competency.

## Introduction

Prematurity is a topic of significant interest for health professionals, as infants born before the 37th gestational week are at a higher risk of falling ill and dying than term neonates; therefore, the care and assistance provided to them represent an important challenge^(1)^. In addition, it is necessary to consider that premature births expose parent to a complex experience loaded with guilt, anxiety, stress, fear and uncertainty^(2-3)^, not only during birth and hospitalization but also in the transition and home-based care^(2)^.

Care continuity after discharge is a determining factor for the well-being of premature newborns, who require special care for a given period of time to ensure their adequate growth and development^(4)^. For this reason, mothers and fathers alike should acquire diverse knowledge and skills related to care before discharge from neonatal units^(4-5)^, but it is also necessary to assess and include emotional and social aspects that favor and ease home-based care^(5)^.

These aspects reassert the need to expand understanding about the experiences undergone by parents in the discharge preparation process and to integrate it into the design of educational interventions that may allow reinforcing care competency for premature newborns. Consequently, the mixed-methods design was a key aspect of the study. The findings from the first phase (qualitative) guided the intervention design and its theoretical approach. This phase provided elements that helped establish the intervention structure and application modality in the professional practice reality and allowed designing a socially sensitive and pertinent intervention, considering the opinions of mothers and fathers that had undergone the experience. The study objective was to design and assess an intervention targeted at reinforcing parents’ competency in caring for premature newborns.

## Method

### Type of study

**Figure 1-:**
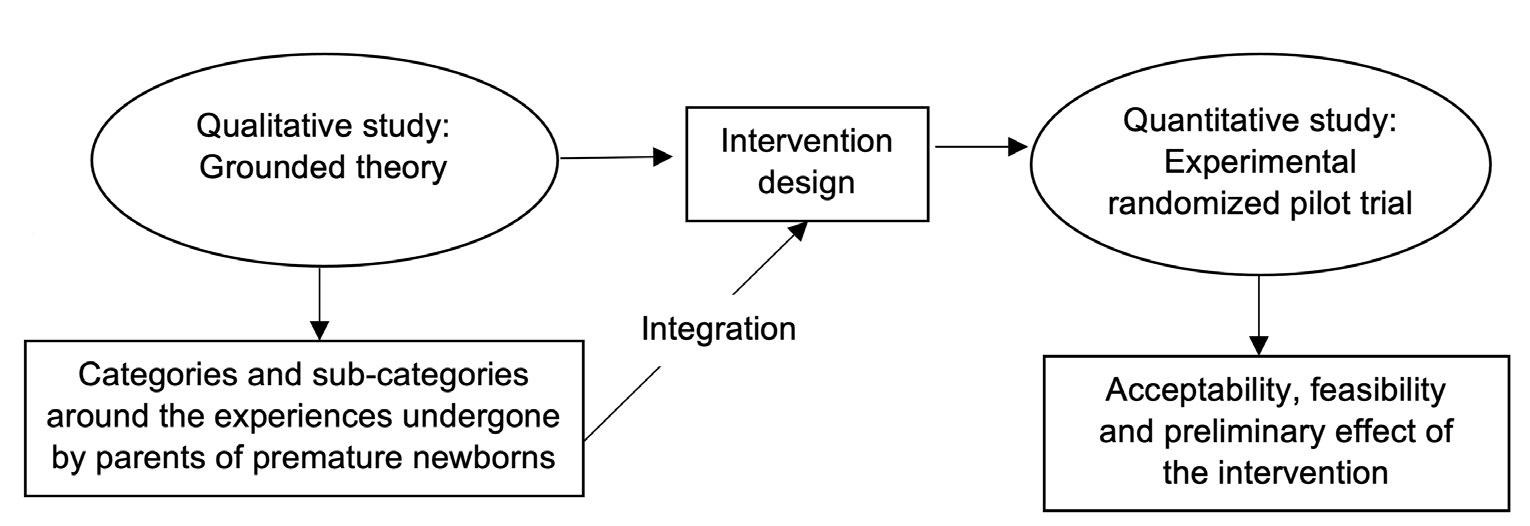
Overall structure showing the mixed-methods design

An exploratory and sequential mixed-methods intervention study where both approaches had the same predominance: QUAL → QUAN^(6-7)^. This type of research proposes a complementarity ratio from the individual perspective of each paradigm. The study is epistemologically grounded on dialectical pluralism, as it favors dialog between different points of view and culturally and socially sensitive research^(8)^ (Figure 1).

Methodological integration was performed based on the connection approach^(6)^. The results from the first phase were integrated into the second one through the intervention design. The qualitative results grounded and guided the intervention, its theoretical perspective, components and operational structure, in addition to helping shape planning of the experimental design procedures for the quantitative component. The integration report during the analysis was made through a narrative^(6)^.

Considering the study methodology and phases, these guides were followed: Consolidated criteria for Reporting Qualitative research (COREQ)^(9)^, CONSORT Statement: Extension to Randomised Pilot and Feasibility Trials^(10)^ and Mixed Methods Appraisal Tool (MMAT)^(11)^; so as to ensure methodological transparency and legitimization of the inferences derived from multiple validity, carefully considering the assumptions that support research process for both qualitative and quantitative components, with the purpose of backing up the mixed-methods study findings^(12)^.

### First phase

#### Type of study

Grounded theory^(13)^.

#### Period

The research was conducted between May 2020 and February 2021.

#### Population and selection criteria

Mothers and fathers of premature neonates born before the 34^th^gestational week, discharged after more than 15 days but at 6 months or less.

#### Exclusion criteria

Mothers or fathers whose children presented congenital malformations or health problems at the interview moment.

#### Participants and definition of the sample

Twelve mothers and four fathers. An initial convenience sampling procedure was implemented following the snowball technique. Theoretical sampling was followed during data analysis, densifying the categories until reaching theoretical saturation, which defined the number of interviews to be conducted^(13)^.

#### Data collection

The interviews were conducted by the lead researcher, a nurse educator with MSc and PhD graduate studies, experience in qualitative research and ample professional experience and sensitivity in terms of neonatal care. The participants were contacted via phone calls and informed about the research objectives, reasons and interests, as well as those of the researcher. After accepting to participate, the dates and times for the semi-structured interviews were scheduled to then conduct them by video calls. None of the individuals invited refused to participate. An interview guide was applied with questions encompassing the core topics inherent to discharge preparation from neonatal units, such as experiences around the way in which the information was received and experiences when getting involved in their newborns’ care in the unit and after discharge, among others. New questions arose as data analysis progressed; these data allowed deepening on some topics along with the theoretical sampling, in order to obtain information about the phenomenon and understand its dimensions. The interviews lasted between 30 and 80 minutes. Each interview was audio-recorded with the participants’ consent. Field notes were taken during each interview to complement the information and enrich the analysis.

#### Data treatment and analysis

The lead researcher transcribed the interviews within 48 hours after they had been conducted. The analysis was performed in a dynamic, non-sequential way by constantly comparing the data^(13)^. Open coding was implemented, identifying incidents and assigning codes. Subsequently, axial coding was used to relate categories and sub-categories and formulate some precise explanations about the phenomenon; finally, the central category was identified through selective coding, which allowed understanding the path undergone in preparing to care for a premature newborn.

In order to ensure rigor of the qualitative research^(14)^ and based on credibility, a verification procedure was implemented during the interviews by means of verbal synthesis or repetitions, so as to ensure that the researcher’s interpretations were consistent with the participants’ testimonies. A triangulation process with the advisor was also performed during the analysis to review and validate the emerging findings and identify possible situations that might exert an influence on the interpretations. In addition, the interviews and results were returned to the participants. As for confirmability, a file that included detailed documentation about the study context, participants, characteristics, procedures and data analysis process was kept, including the audios and transcriptions from the interviews, memoranda, matrices and diagrams. The study processes and details were duly recorded and organized in a file, which will allow other researchers and professionals to evaluate and apply its results in similar contexts and populations to the ones researched, therefore enabling their transferability.

### Second phase

#### Type of study

A Randomized Pilot Study (RPS) with a parallel design and no masking^(15)^. This type of study involves all the processes inherent to clinical trials, but with a smaller sample; they are focused on establishing the feasibility, acceptability and preliminary effect of an intervention^(16)^.

#### Period

The total recruitment and follow-up time was 6 months, ending in July 2022.

#### Population and selection criteria

Mothers of premature infants born at the 34^th^ gestational week at the most and hospitalized in a Neonatal Intensive Care Unit (NICU) for at least 48 hours after admission.

#### Exclusion criteria

Mothers with previous experience in caring for premature newborns and/or children with some congenital malformation.

#### Sample

No formal sample size calculations are usually made in RPSs; however, there are recommendations about a minimum number of participants, which is estimated at between 10 and 12 per group^(17)^. Thus, a total of 28 mothers took part, 14 in each group.

#### Randomization

A simple randomization process was performed in EPIDAT 4.2. The sequence generated was totally alien to the researcher and sealed opaque envelopes were handled. All the intervention sessions were in charge of the lead researcher. The study intervention was applied to one group, whereas the other one was offered the usual discharge preparation. The response variables were measured at three moments: before the intervention (baseline); close to discharge (1 day before discharge or at discharge); and one week after discharge (7-9 days). The main response variable was “competency in caring for premature newborns at home”. The following were considered as secondary response variables: weight gain, total hospitalization days and exclusive breastfeeding, among others.

#### Data collection

*Ad hoc* instruments were used to measure the variables of interests; in turn, the CUIDAR Ma-Pre (adapted from the CUIDAR scale designed and validated in Colombia to assess care competency, understood as a person’s or their caregiver’s ability to perform their care tasks at home) was employed for the main response variable^(18)^. Although the scale has already been applied in various contexts, it has not yet been used in mothers of premature newborns, reason why it was to necessary to adapt it, resulting in the CUIDAR Ma-Pre version. For this new version, a semantic adequacy process was implemented, in addition to an experts’ evaluation where a Content Validity Index (IVC) of 0.816 was obtained. Agreement among the evaluators was moderate, with a Fleiss Kappa index of 0,51. After empirically applying the instrument to 207 mothers, an exploratory factor analysis was performed, determining suitability of a sample with Kaiser-Mayer-Olkin statistics=0.859 and a Bartlett’s sphericity test value of 2,953.9 (p=0,00). After proposing various scenarios, a model with 7 dimensions and 33 items explained 57.9% of the variance, with a Cronbach’s alpha of 0.852. The goodness of fit tests allowed establishing that the model presented good fit, through statistical signification: χ^2^=0.01, CFI=0.92, BIC≥10 and RMSEA=0.05^(19)^. The dimensions included in the CUIDAR Ma-Pre instrument are as follows: *Actúa*(Acts), *Afronta*(Faces), *Vínculo*(Bond), *Apoyo social*(Social support), *Conocimiento general*(General knowledge), *Unicidad*(Uniqueness) and *Conocimiento específico*(Specific knowledge).

#### Data treatment and analysis

The data were stored and analyzed in the SPSS software, version 27.0. Central tendency, dispersion and position measures were calculated for the quantitative variables and the qualitative ones were described by means of absolute and relative frequencies. In order to compare the groups in their baseline conditions regarding the variables of interest, hypothesis tests for independent groups were performed, in addition to Student’s t or Chi-square tests according to data distribution and nature of the variables. Statistical signification with p-values below 0.05 and 95% confidence was assumed in all cases. The Student’s t test was used when comparing the caregivers’ competency scores and in the analysis of the differences according to each measurement moment. An ANOVA-type model was explored to determine if any variable of theoretical or statistical interest intervened in them.

#### Ethical aspects

The study was endorsed by the Research Ethics Committees of the Nursing School belonging to *Universidad de Antioquia* (No. CEI-FE 2020-02) and of the health institution where the study was conducted (No. CI-HGM 10-0709).

### Qualitative phase results

The participants were 12 mothers and 4 fathers: 5 of them had university studies, 4 had technical education, 5 had High School and 2 had Elementary School. There were 5 parents of twins. The newborns’ minimum and maximum weights were 625 g and 2,135 g, respectively. Their minimum and maximum gestational ages were 26 and 34 weeks, respectively.

The results indicate that discharge preparation and reinforcement of the parents’ competency in caring for their newborns at home are complex and dynamic processes. Four categories emerged: 1) Facing premature births, 2) Experiencing the prematurity routine in Neonatal Units, 3) Self-empowerment to care for premature newborns, and 4) Caring for premature newborns at home.

### Facing premature births

It indicates that premature births are an unknown situation for parents, who must face a new and complex reality that differs from their expectations. An ideal birth means having their child, taking them home and assuming the parenthood challenges in their environment, but premature births are a different experience. This category is comprised by the following sub-categories: “Understanding the premature birth causes living an unimaginable situation”, “Living between fears and hope” and “Getting to know premature newborns”. Some of the testimonies illustrate so: *It was all very complex and surprising, I’d never imagined having a premature child*(P3). *It was all so hard, because you imagine having your child, then going home and everything’s gonna be OK, but it was different for us*(P5). When facing premature births, it is fundamental to have due monitoring and information easing this process.

### Experiencing the prematurity routine in Neonatal Units

This category includes the following sub-categories: “Going through ups and downs”, “Interacting with premature newborns”, “Kangarooing premature newborns” and “Breastfeeding and Kangarooing in the unit”. It represents the everyday and common experiences inherent to the process of caring for their children. Assuming motherhood and fatherhood in this context implies uncertainty, fear and pain, as well as a significant need for support, monitoring and information: *We’re fine today, we’re not fine tomorrow, you just don’t know with them, every premature baby is like that and you’re full of fears because their evolution is very uncertain*(P3). In the prematurity routine, parents initiate interaction and contact with their newborns in a limited way: skin-to-skin contact following the Kangaroo method is stated as a specific care and interaction modality: *I put it to my chest and it was as if we were interpenetrating ourselves in our souls, as if we were meeting again*(P5).

This category accounts for the complexity of the parents’ experiences in neonatal units. These initially unknown spaces become their reality.

### Self-empowerment to care for premature newborns

It is a phase marked by care appropriation and empowerment that favors the hospital-home transition and reinforces the competency to care for premature newborns. The parents not only learned about the care they should provide to their children but also overcame fears and ceased to be mere receptors to appropriate the role: *I wanted to do everything, because they’re my children, then I wanted to learn*(P5); *towards the future because we can’t go back in time, it’s just empowering ourselves and accepting the situation*(P11).

Empowerment emerges from experience as a resource that allows them to face difficulties undergone in the process and gain strength to assume the care of their children. It is comprised by the following sub-categories: “Learning to care for premature newborns”, “Recognizing my capabilities and”Experiencing discharge preparation”.

### Caring for premature newborns at home

This category includes the following sub-categories: “The discharge moment”, “Coming home”, “Receiving support” and “Follow-up after discharge”. Care at home faces parents with having to independently assist their children, away from the hospital setting. Returning home is a new beginning: discharge from the unit faces them with new emotions and with an unknown reality. Although the parents have in fact prepared for this moment, the complexity inherent to the experience extends to their homes and faces them with mixed emotions and fear regarding possible complications: *I was afraid that something bad could happen to him suddenly at home, that he wouldn’t be able to breathe*(P9).

Returning home faces parents with actual and genuine motherhood and fatherhood, not conditioned by the context and protocols in neonatal units: *You’re freer to be a mom already at home*(P1). Family support is fundamental, as premature newborns present high care requirements. Follow-up in the Kangaroo plan allows connecting with health professionals and support for home-based care.

### Empowerment as a central category in preparing to care for premature newborns

Empowerment was a determining factor in strengthening the care competency and the hospital-home transition. It has to do with the conditions and events that allowed the participants to acknowledge their capabilities, believe in themselves, overcome fears and get actively involved in their newborns’ care in the neonatal unit. In addition, it mobilized strength and determination for care. The greater the participation in the care preparation process, the greater the empowerment. The information received, the support, the ups and downs experienced and the trusting relationship with the Nursing personnel contribute to empowerment and characterize an individual perspective in the process.

### Mixed-methods integration: Design of the Acciones para un Cuidado Neonatal Empoderado (ACUNE) intervention (Actions for Empowered Neonatal Care)

The qualitative component results were connected to the quantitative phase when guiding the intervention design, its theoretical approach and structure, as well as the experimental design procedures. The integration made based on the intervention design can be identified inFigure 2.

**Figure 2-:** Connection between the qualitative findings and the intervention and quantitative phase

**Qualitative finding**	**Connection with the intervention design and the experimental phase procedures**
**Central category: Self-empowerment to care for premature newborns.**	Theoretical grounds for the intervention: Self-empowerment to care for premature newborns.
**Category: Facing premature births.** It states the complexity of the situation at the initial moment; everything is unknown, the parents need support and guidelines to place themselves in the situation and context. **Category: Experiencing the prematurity routine in neonatal units.** It has to do with adapting to an unknown environment, where help is required from Nursing personnel to get involved in caring for their newborns and trusting in their capabilities. **Category: Caring for premature newborns at home.** It is based on the need to mobilize and undergo a new experience in the process, where new fears arise in the face of the unknown and regarding their care competency.	**Session 1.** First hospitalization week: *Recognizing prematurity and the NICU environment* . The content and delivery modality are guided by the parents’ experience. The instruments and intervention are applied after the first day since, according to the parents’ experience, it is not possible to process them the first day. **Session 2.** From the second to the third hospitalization week (after the first session and before the third one): *Getting to know the singularities of caring for premature newborns* . It includes contents about caring for premature newborns and direct contact by means of the Kangaroo method, feeding, nasal hygiene and other practical activities. It is based on monitoring and on fostering safety and self-confidence. **Session 3** : Within 2 days before discharge: *Preparing to go home.* It includes information and monitoring to favor the transition. It involves the family and is targeted at personal and social resources for empowerment. It includes aspects related to alert signs, administrative processes and attendance to the follow-up program.
**Central category: Empowerment for** **care**	Booklet design, interventionists’ manual, empathetic relationship and support in practical care activities, including demonstration and simulation ones. Definition of the proper moments to apply instruments and questionnaires, as well as to deliver the intervention.

#### Theoretical grounds for the intervention

Empowerment is a central aspect to reinforce the care competency in the case of premature newborns, as it favors discharge preparation and the hospital-home transition. The ACUNE intervention is grounded on the Theory of Health Empowerment^(20)^, which considers human beings as a whole with their environment in everyday life and in their health experiences; characterized by self-organization patterns, diversity and innovating changes, in addition to recognizing individual values and points of view about health. The theory identifies that empowerment emerges from personal and socio-contextual resources^(20)^. The former reflect unique characteristics inherent to each individual and the latter include social support and from health personnel. From this perspective, it is reasserted that empowerment is a dynamic health process that emphasizes the possibility of intentionally taking part in a process of self-change and to modify the environment, recognize patterns and take on a commitment to well-being resources. Empowerment eases conscientious participation in health care process decisions^(20-21)^. The empowerment described by the parents coincides with the theoretical perspective.

#### Description of the ACUNE intervention

The proposal set forth by Sidane and Braden^(22)^ was followed to specify the intervention and its structure. The ACUNE intervention aims at reinforcing the competency to care for premature newborns at home, through an empowerment intervention during discharge preparation from neonatal units. It includes three components: Cognitive, Social and Behavioral.

The Cognitive component aims at strengthening personal resources for health empowerment and considers the knowledge and skills mothers should acquire to care for their premature newborns and which constitute a personal resource for empowerment. The Social component seeks to favor recognition of the contextual social resources for empowerment. It includes activities seeking to ease recognition of support networks and health care services. Finally, the Behavioral component aims at promoting conscientious participation in the discharge preparation process and reinforcing the parents’ competency to care for their premature newborns at home.

The intervention is delivered face-to-face; simulation or direct care activities are performed according to each child’s clinical condition. Spaces to ask questions and express emotions and needs regarding the situation are created during the sessions. Between each session and the next, visits are made to clear the mothers’ doubts and they are encouraged to take part in the educational activities offered by the health institution. The intervention also includes a monitoring session on the day the participants enter the “Kangaroo program”, in order to favor the transition to the new care space.

It includes an educational booklet as an informative and guiding tool. Each section is complemented by blank spaces for the mothers to describe their emotions, reflections, questions or needs. It also contains tips for motivation and to recognize personal and social resources for empowerment. The component that deals with the transition includes a checklist that mothers complete by assessing their knowledge and skills for care and which leads them to ask for support according to their own identification of needs and resources. The intervention and material were assessed by 3 experts in the area with PhD training in Nursing and experience in intervention designs, reaching consensus in terms of coherence, suitability and relevance. Their suggestions were welcomed before initiating the assessment process.

### Quantitative phase results

**Figure 3-:**
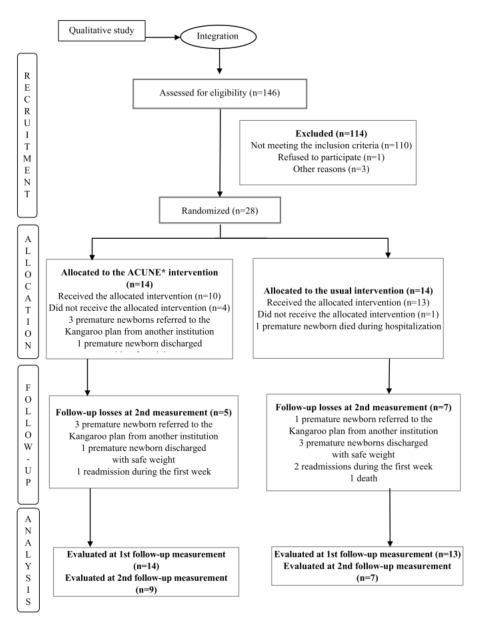
Flowchart corresponding to the study participants

Note: Adapted^10^.

A total of 146 admissions were recorded during the recruitment period: 32 (21.9%) of them met the inclusion criteria and the recruitment percentage was 87.5%. Of all the mothers from the Intervention Group, 4 failed to finish the monitoring session due to administrative issues, which suggests the need to adapt the protocol to minimize losses. Figure 3 shows the flowchart corresponding to the pilot study participants; in turn, Table 1 presents a description of the sociodemographic variables per study group, where comparability of the groups can be seen.

*ACUNE = *Acciones para un Cuidado Neonatal Empoderado*

**Table 1-:** Sociodemographic variables by study groups. Medellin, Antioquia, Colombia, 2021

Variable	Control Group n*=14	Intervention Group n*=14	Sig ^†^
Mother’s age	25.14± *7.44*	26.93±5.78	0.485 ^‡^
Birth weight, in grams	1,327.64±279.31	1,695.64±213.60	0.001 ^‡^
Gestational age at birth	31.21±1.42	32.12±1.16	0.076 ^‡^
Number of children	2.93±2.303	2.14±1.027	0.701 ^§^
Care competency	81.43±15.37	80.71±16.85	0.908 ^‡^
**Socioeconomic strata**	4	6	0.301 ^||^
Stratum 1	2	3	
Stratum 2	0	1	
Stratum 3	0	1	
Stratum 4	8	3	
Does not know			
**Social Security regime in health**	8	7	0.177 ^||^
Subsidized	0	3	
Contributive	6	4	
Linked			
**Schooling level**	4	0	0.179 ^||^
Incomplete Elementary School	2	1	
Complete Elementary School	2	2	
Incomplete High School	6	8	
Complete High school	0	2	
Technical level	0	1	
University studies			
**Area of residence**	10	11	0.663 ^||^
Urban	4	3	
Rural			
**Nationality**	10	9	0.686 ^||^
Colombian	4	5	
Venezuelan			
**Marital status**	4	0	0.094 ^||^
Single	1	1	
Married	9	13	
Consensual union			
**First child**	7	9	0.445 ^||^
Yes	7	5	
No			
**Multiple birth**	2	3	0.622 ^||^
Yes	12	11	
No			
**Weight for gestational age**	4	2	0.648 ^||^
Low	10	12	
Adequate			
**Has support from the child’s father**	11	14	0.067 ^||^
Yes	3	0	
No			

*n = Sample; ^†^Sig = Statistical significance; ^‡^t = Student’s t test; ^§^U = Mann-Whitney’s U test; ^||^Chi^2^ = Chi^2^ test

As for the preliminary effect, by applying a Student’s t test for independent samples in the comparison between groups, it was possible to determine that care competency improved in both, although the difference was not significant. Baseline measurement: Control (n=14) 81.43±15.37 vs Intervention (n=14) 80.71±16.85 p=0.908; measurement at discharge: Control (n=13) 117.08±6.103 vs Intervention (n=14) 121.14±7.156 p=0.126; and measurement one week after discharge: Control (n=7) 112.56±6.241 vs Intervention (n=9) 118.67±9.421 p=0.163.

In turn, by means of a Student’s t test for related samples in the intra-group comparison, it was identified that the competency level at hospital discharge increased in relation to the baseline measurement, finding a statistically significant difference in favor of the intervention, reason why Cohen’s d was calculated to determine the effect size: Control (n=13) baseline 82.15±15.74 vs at discharge 117.08±6.10 p=0.000, Cohen’s d 12.72; Intervention (n=14) baseline 80.71±16.85 vs at discharge 121.14±7.15 p=0.000, Cohen’s d 18.55.

This same trend was also identified in the measurement made one week after discharge when compared to the baseline one: Control (n=7) baseline 86.71±17.14 vs one week after discharge 112.57±6.24 p=0.004, Cohen’s d 15.302; Intervention (n=9) baseline 81.22±16.10 vs one week after discharge 118.67±9.421 p=0.000, Cohen’s d 16.853.

The following was found for the difference between the discharge moment and one week after it: Control (n=7) at discharge 117.43±7.829 and one week after discharge 112.57±6.241 p=0.192 vs Intervention (n=9) at discharge 121.33±7.365 and one week after discharge 121.14±7.156 p=0.335. However, the difference was not statistically significant.

As for the effect exerted by the ACUNE intervention on important secondary variables, statistically significant differences were only found between the study groups in terms of hospitalization days in basic care: Control (n=13) 11.38±10.054 vs Intervention (n=13) 4.14±2.656 p=0.025.

The possible interaction between the variables that might intervene in the score differences was explored. To such end, a repeated measures ANOVA test based on the three measurements corresponding to the caregivers’ competency was applied, finding no statistically significant differences that would indicate that some of them might exert an influence on the care competency result.

Finally, it was identified that the ACUNE intervention presents adequate acceptability. All the mothers pointed out that the intervention seemed important, useful, clear and pertinent to them. In relation to the intervention structure, it was found that 50% of the mothers thought that the sessions were too brief and that 57.1% believed that the number of sessions was insufficient. During the acceptability assessment, the participants were also asked about the booklet and its usefulness, pertinence, presentation and clarity: all the mothers assessed it as “useful” or as “very useful”. Most of the mothers assessed its presentation, clarity and content as excellent.

Figure 4 presents the data connection in a joint display, where the qualitative findings seek to explain and complement the statistically significant quantitative ones obtained in the second phase to generate the data meta-inferences.

**Figure 4-:** Joint display showing the data and meta-inferences

**Quantitative results** **Care competency(intra-group)**	**Qualitative results** **Self-empowerment for care: Testimonies**	**Meta-inferences**
**Control Group (n=13)** Baseline measurement 82.15±15.74 At discharge 117.08±6.10 p=0.000 **Intervention Group (n=14)** Baseline measurement 80.71±16.85 At discharge 121.14±7.15 p=0.000	*[…] I believe that you have to empower yourself, they’re my children, this is what I’ll have to do with them and I have to learn with no fears […]* (P5) *.* *[…] Moving on because we can’t go back in time, it’s just empowering ourselves and accepting the situation as it is; you only learn when you understand that, some nurses used to tell me that I was able to do it, that I should trust in my capabilities, that if other moms could do it, I could do it too; and that was what most helped me […]* (P11) *.*	Preliminary, the intervention with empowerment as its central focus showed that it improves care competency, in line with the experiences undergone by the parents, who reported that empowerment was essential to learning how to care for their children. Parents need more than knowledge, and it is necessary to support them in overcoming fears when facing their children’s weakness and to get involved in care within neonatal units. An empowering Nursing intervention exerts a positive effect on care competency.
**Control Group (n=7)** Baseline measurement 86.71±17.14 One week after discharge 112.57±6.24 p=0.004 **Intervention Group (n=9)** Baseline 81.22±16.10 One week after discharge 118.67±9.421 p=0.000	*[…] I used to say that if they let me take her home it’s because she’s OK and they see that she can breathe by herself and because I’m going to be able to take care of her […]* (P8). *[…] What we’ve been waiting for is finally going to happen, being prepared to go home […]* (P5) *.* *[…] we were already going home, we were with the nurses there instead and it was but pressing a button that the problems were solved, you do get afraid here at home […]* (P9).	The intervention was designed to promote empowerment after hospital discharge, and the experimental study results showed certain effect in favor of the intervention in that sense. Although the results are preliminary, they point to the convenience of including this theoretical approach in discharge preparation processes. On the other hand, the results allow identifying that the competency level at discharge is higher than the one recorded one week after it, which indicates that the first week at home is critical for parents, who face the reality inherent to independent care, an aspect that was reported in the interviews.
**Hospitalization days in basic** **care:** Control (n=13) 11.38 ± 10.054 Intervention (n=13) 4.14±2.656 p=0.025 As for acceptability, 50% of the mothers thought that the sessions were too brief and 57.1% believed that the number of sessions was insufficient.	*[…] I already wanted to go home, I already felt prepared, I’d done it before, I’d done it there in the unit, I wanted to leave quickly, but because I already knew how to care for my son and felt confident […]* (P5). *[…] I used to say “I’ll do it!” to everything. And they asked me: “Can you?”. I answered “No”, but I have to learn, then what I’m gonna do when I take him home? I won’t have you, I want to learn here, learn from you all and the more you teach me, the better […]* (P1).	Preliminarily, the results show that the intervention reduces the number of hospitalization days in basic care, the assistance context prior to discharge. As they have a sense of empowerment, the mothers feel self-confident and show their competency when assessing this discharge criterion. Considering empowerment as a differential element, in addition to the material and the delivery modality, the mothers expect more sessions and time in this sense since, based on their experience, better direct monitoring generates greater self-confidence for care.

## Discussion

The qualitative findings ratify the complexity and multi-dimensionality inherent to having a premature child^(3)^. Discharge preparation is not an isolated process; it is combined within the experiences around birth, hospitalization in neonatal units and caring for newborns at home. The participants do not define any beginning or end in terms of their preparation to care for their children, reasserting it as a continuous process that starts at admission to the neonatal unit and simply does not end at hospital discharge^(3,23)^.

The participants’ experience begins with their child’s premature birth and with the life events that face them with an unexpected reality. Subsequently, they get familiar with the neonatal unit’s routine where they bond with their child, empower themselves and get prepared for home-based care. Finally, they go home and face a reality where care, support and follow-up programs play a fundamental role in their experience. These findings are consistent with other qualitative studies where experiences undergone by parents of premature newborns have been explored, finding that, as in the case of the current research, hospitalization and the hospital-home transition represent a complex and dynamic path towards competent home-based care^(23-25)^. Such conclusions were integrated into the intervention design, its application and the very procedures of the experimental study, finding that the results are consistent and confirmed through the mixed-methods integration. As was the case with the parents’ experiences where they stated that when the Nursing personnel helped them feel capable and empowered, they managed to take better care of their children, the intervention targeted at empowerment achieved a positive preliminary effect in its favor.

Neonatal units’ routines are marked by the need for information about the children’s health and regarding the assistance dynamics inherent to the context, factors already identified in other studies^(22-24)^. This points to the pertinence of including information for parents to get familiar with neonatal units, as they represent unknown and stressful environments^(24-26)^. This aspect was fundamental in the intervention and the core axis for the first session, generating a pertinence perspective in the initial approach to the educational process.

Based on the participants’ experience, empowerment is central in reinforcing the competency to care for their children at home, as it emerges from assuming the parental role and feeling responsible for such care. These events mobilize strength and determination to getting involved in care and overcoming fears. The information and the support from the Nursing personnel and the family contribute to empowerment, which begins in the neonatal unit and gains strength at discharge. The need for empowerment has already been present in other studies, which reassert that supporting parents’ active participation in the care of their children in these units is an empowerment mediator, which contributes to safety and self-confidence for the transition^(27-28)^. As it focuses on empowerment, the ACUNE intervention is centered on the parents’ experiences and uses them as a starting point to verify that this approach exerts a positive result on the their experience and care ability.

Empowerment has been addressed in health education as part of the trend towards participatory assistance and care^(29-30)^. This reasserts the meaning of empowerment within the parents’ discharge preparation processes from neonatal units and favors integration of the empowerment theory into the ACUNE intervention.

According to the theory, health empowerment is a relational process that emerges from acknowledging each individual, their personal resources and social context^(19-20)^. This principle is in line with the participants’ experiences and emerges as a central aspect of the ACUNE intervention, whose preliminary assessment indicates that it is viable and acceptable and that it can exert a positive effect on parents’ competency to care for premature newborns. The intervention proved to be viable in terms of physical and human resources. Its simple structure enables it to be delivered to mothers as planned. Measuring the variables of interest was deemed viable and the instruments were seamlessly applied during the entire study.

No statistically significant differences were found when analyzing the home-based care competency results between the groups; however, it was identified that the competency scores increased in both groups, being higher in the Intervention one. The mothers from both groups were offered the usual intervention; however, those from the Intervention Group were also applied the ACUNE intervention, which unlike the habitual one, has a defined structure linked to an empowerment disciplinary theoretical framework.

When evaluating the intra-group effect, it was possible to notice favorable changes in both groups according to the measuring moments, identifying statistically significant differences in this analysis. This preliminary result indicates that the intervention might improve the mothers’ competency to care for their premature newborns at home, suggesting that it is pertinent to conduct the randomized study at a larger scale.

When comparing the results to other studies that have sought to determine the effect exerted by the empowerment approach, it is important to acknowledge that the evidence asserts it has favorable effects. A recent study conducted with 80 mothers (40 in each group) found that a counseling intervention targeted at mothers of premature newborns presented a statistically significant difference in the self-esteem and stress mean scores: 9.52±3.22 and 16.75±6.39 (p<0.001) and 154.65±32.15 and 61±10.98 (p<0.001) respectively. In terms of stress, the most important reduction was identified in the “Breastfeeding infant’s aspect and behavior” dimension (Cohen’s d=3.146)^(29)^. This finding reasserts the coherence found between the ACUNE intervention and the evidence, as information related to this topic was included given the first phase findings where, through the parents’ experiences, it was identified that their premature newborns’ initial aspect was shocking and generated fears during care and direct contact.

An outstanding aspect is that, although the measurements at discharge and one week after it in the Intervention Group were higher than in the Control Group, a reduction was identified in the scores after discharge, related to the fact that the first week of the hospital-home transition is critical and to the parents facing new challenges that may question their care capabilities, with the possibility that their self-confidence is reduced^(28)^.

In the current analysis, it is necessary to consider that the results in relation to the effect are preliminary. As the study sample size is small, it holds limited statistical power, a fact to which the high number of losses is added in this particular case. These aspects can be somehow expected in pilot studies, reason why the chances of finding differences and performing another type of analysis are limited. Therefore, although guiding, interpretation of the results is precautious and non-conclusive regarding the effect; in addition, it allows knowing aspects related to viability and acceptability of the intervention, optimizing time and resources when conducting the Randomized Clinical Trial.

The intervention acceptability was good and the main adjustment suggested after the pilot test had to do with viability of the follow-up to which the mothers referred to other institutions were subjected. In this sense, it is necessary to ensure and clarify access to the mothers to take after-discharge measurements, using online means to reduce the number of losses.

These results reassert that health empowerment is a theoretical perspective that allows improving the mothers’ capabilities and self-confidence to care for their premature newborns. It arises from the parents’ experience and confirmed when assessing an intervention based on this approach, which turns out to be socially sensitive, validated by the parents’ experiences and preliminary confirmed by means of an experimental study that opens up a number of possibilities for new phases to confirm its effect and confers value and meaning to the mixed-method research, which involves the opinions of those taking part in the phenomena of interest for the discipline.

The preliminary assessment of the ACUNE intervention indicates that it might exert a positive effect on parents’ competency to care for premature newborns at home. The results are preliminary and non-conclusive, but do serve as a guide as for the convenience of conducting the main study.

The pilot study sample size is considered as a limitation. Although it is not generated by means of formal calculations and the scope only serves as a guide for this type of study, larger samples might more closely represent the actual effect. However, designing an intervention that emerges from the parents’ opinions and the preliminary confirmation of empowerment as a theoretical approach that improves parents’ competency to care for premature newborns are acknowledged as strengths based on its mixed-methods perspective.

## Conclusion

The experiences undergone by mothers and fathers in developing and reinforcing their competency to care for premature children are complex and dynamic. Empowerment is a central aspect that favors care competency, self-confidence and the hospital-home transition. Participation in the care provided, family support and monitoring and interaction with Nursing personnel are essential for empowerment. Designing a Nursing intervention to reinforce parents’ competency in caring for premature newborns based on the empowerment approach has theoretical grounds that allow applying it in the practice; in addition, there is empirical evidence supporting the convenience inherent to this type of interventions. Having integrated the qualitative results into the intervention design favored its social sensitivity and proximity to the mothers’ reality and to the Nursing professional practice.
