# Supplementary material for: Design and assessment of an intervention for parents of premature newborns: a mixed-methods study
Source: Rev Lat Am Enfermagem. 2026 Feb 2;34:e4747. doi: 10.1590/1518-8345.7791.4747 (PMC12863659; doi:10.1590/1518-8345.7791.4747)
Supplement: Translation

# Translation: Desenho e avaliação de uma intervenção para pais de bebês prematuros: um estudo de métodos mistos*

**DOI:** 10.1590/1518-8345.7791.4748

**Authors:** Sandra Patricia Osorio-Galeano, Angela María Salazar-Maya

**Affiliations:** 1 Universidad de Antioquia, Facultad de Enfermería, Medellín, Antioquia, Colômbia; 2 Universidad CES, Facultad de Enfermería, Medellín, Antioquia, Colômbia

**Keywords:** Enfermagem Neonatal, Recém-Nascido Prematuro, Pais, Unidades de Terapia Intensiva Neonatal, Empoderamento, Pesquisa em Enfermagem

## Abstract

desenhar e avaliar uma intervenção para fortalecer a competência no cuidado de filhos prematuros.

trata-se de um estudo de intervenção de métodos mistos, exploratório e sequencial. A teoria fundamentada foi aplicada na primeira fase. Participaram 12 mães e 4 pais. Na segunda fase, um estudo-piloto randomizado foi implementado com 14 mães em cada grupo. A principal variável de resposta foi a competência para o cuidado. A escala CUIDAR Ma-Pre foi administrada na medição basal e em duas medições de acompanhamento.

quatro categorias emergiram: Enfrentando o parto prematuro, Vivenciando o cotidiano da prematuridade na Unidade Neonatal, Empoderando-se para o cuidado do bebê prematuro e Cuidando do filho prematuro em casa. Empoderando-se para o cuidado do bebê prematuro foi a categoria central. Esses resultados foram integrados ao desenho da intervenção. A avaliação encontrou diferenças a favor da intervenção. A diferença não foi estatisticamente significativa entre os grupos, mas sim intragrupo (Grupo de Controle n=13, basal 82,15; na alta 117,08, p=0,000 vs. Grupo de Intervenção n=14, basal 80,71; na alta 121,14, p=0,000).

o empoderamento foi essencial para a experiência; uma intervenção com essa abordagem é conveniente, e sua avaliação preliminar sugere que ela pode melhorar a competência para o cuidado. É pertinente um estudo em maior escala. ClinicalTrials.gov. Identificador: NCT05005988.

## Destaques

**(1)** O empoderamento é fundamental na experiência de cuidar de crianças prematuras. **(2)** É conveniente desenhar uma intervenção com base na experiência dos pais. **(3)** A abordagem teórica do empoderamento é aplicável às intervenções educacionais. **(4)** Uma intervenção direcionada aos pais de bebês prematuros é viável e aceitável. **(5)** O empoderamento pode fortalecer a competência para o cuidado.

## Introdução

A prematuridade é um tema de grande interesse para os profissionais de saúde, visto que crianças nascidas antes de 37 semanas de gestação apresentam maior risco de adoecer e morrer do que crianças nascidas a termo, portanto, seu cuidado e atenção representam um grande desafio^(1)^. Além disso, é preciso considerar que o nascimento prematuro expõe os pais a uma experiência complexa, repleta de culpa, ansiedade, estresse, medo e incerteza^(2-3)^, não apenas durante o parto e a hospitalização, mas também durante a transição e o cuidado domiciliar^(2)^.

A continuidade dos cuidados após a alta é crucial para o bem-estar dos recém-nascidos prematuros, que por um tempo irão precisar de cuidados especiais para garantir seu crescimento e desenvolvimento adequados^(4)^. Por isso, tanto mães quanto pais devem adquirir conhecimentos e habilidades para o cuidado antes da alta da unidade neonatal^(4-5)^, mas também é necessário valorizar e envolver aspectos emocionais e sociais que favoreçam e facilitem o cuidado domiciliar^(5)^.

Esses aspectos reafirmam a necessidade de ampliar a compreensão das experiências dos pais no processo de preparação para a alta e integrá-las ao desenho de intervenções educacionais que fortaleçam a competência no cuidado de crianças prematuras. Assim, um aspecto fundamental do estudo foi o desenho de métodos mistos. Os achados de uma fase qualitativa inicial orientaram o desenho da intervenção e sua abordagem teórica. Essa fase forneceu elementos que ajudaram a estabelecer a estrutura e a implementação da intervenção na realidade da prática profissional e permitiram o desenho de uma intervenção socialmente sensível e adequada, considerando as vozes de mães e pais que vivenciaram a experiência. O objetivo do estudo foi desenhar e avaliar uma intervenção para fortalecer a competência no cuidado de filhos prematuros.

## Método

### Tipo de estudo

Estudo de intervenção sequencial exploratório de métodos mistos em que ambas as abordagens tiveram igual predominância QUAL → QUAN^(6-7)^. Este tipo de pesquisa propõe uma justificativa para a complementaridade a partir da perspectiva individual de cada paradigma. O pluralismo dialético sustenta epistemologicamente o estudo, pois favorece o diálogo de perspectivas e uma pesquisa cultural e socialmente sensível^(8)^ (Figura 1).

**Figura 1-:** Estrutura geral do desenho de métodos mistos

A integração metodológica foi alcançada por meio da abordagem de conexão^(6)^. Os resultados da primeira fase foram integrados à segunda por meio do desenho da intervenção. Os resultados qualitativos informaram e orientaram a intervenção, sua perspectiva teórica, componentes e estrutura operacional, e ajudaram a moldar o planejamento dos procedimentos do desenho experimental do componente quantitativo. A integração foi relatada durante a análise por meio da narrativa^(6)^.

Considerando a metodologia e as fases do estudo, foram seguidas as seguintes diretrizes: *Consolidated criteria for Reporting Qualitative research* (COREQ)^(9)^, *CONSORT Statement: Extension to Randomised Pilot and Feasibility Trials* ^(10)^ e *Mixed Methods Appraisal Tool* (MMAT)^(11)^ para garantir a transparência metodológica e a legitimação das inferências derivadas da validade múltipla, considerando cuidadosamente os pressupostos que sustentam os processos de pesquisa dos componentes qualitativos e quantitativos, com a intenção de apoiar os resultados do estudo de método misto^(12)^.

### Primeira fase

#### Tipo de estudo

Teoria fundamentada^(13)^.

#### Período

A pesquisa foi realizada entre maio de 2020 e fevereiro de 2021.

#### População e critérios de seleção

Pais e mães de bebês prematuros nascidos antes das 34 semanas de gestação, com alta hospitalar superior a 15 dias e inferior ou igual a 6 meses.

#### Critérios de exclusão

Pais cujos filhos tinham malformações congênitas ou que apresentassem problemas de saúde no momento da entrevista.

#### Participantes e definição da amostra

Doze mães e quatro pais. Foi realizada uma amostragem inicial por conveniência e bola de neve. Durante a análise dos dados, seguiu-se uma amostragem teórica, que densificou as categorias, até atingir a saturação teórica que determinou o número de entrevistas a serem realizadas^(13)^.

#### Coleta de dados

As entrevistas foram conduzidas pela pesquisadora principal, enfermeira, docente, com mestrado e doutorado, experiência em pesquisa qualitativa e ampla experiência profissional e sensibilidade para o cuidado neonatal. Os participantes foram contatados telefonicamente, sendo informados sobre os objetivos, os motivos da pesquisa e os interesses da pesquisadora. Após a concordância em participar, foi agendada data e horário para a entrevista semiestruturada, realizada por videochamada. Nenhum dos convidados se recusou a participar. Foi utilizado um roteiro de entrevista com perguntas que abordavam tópicos centrais relacionados à preparação para a alta da unidade neonatal, às vivências sobre o modo como receberam informações, às experiências de participação no cuidado do filho na unidade e cuidados pós-alta, entre outros. À medida que a análise dos dados avançava, novas questões emergiram, permitindo-nos aprofundar alguns tópicos paralelamente à amostragem teórica, obtendo informações sobre o fenômeno e compreendendo suas dimensões. A duração das entrevistas foi variada, oscilando entre 30 e 80 minutos. Cada entrevista foi gravada em áudio com a permissão dos participantes. Anotações de campo foram feitas durante cada entrevista para complementar as informações e aprimorar a análise.

#### Processamento e análise de dados

As entrevistas foram transcritas pela pesquisadora principal em até 48 horas após sua realização. A análise foi realizada de forma dinâmica, não sequencial, por meio da comparação constante dos dados^(13)^. Foi realizada a codificação aberta, com identificação dos incidentes e atribuição de códigos. Em seguida, utilizou-se a codificação axial para relacionar categorias com subcategorias e formar explicações precisas sobre o fenômeno e, por fim, por meio da codificação seletiva, identificou-se a categoria central, que permitiu compreender a trajetória vivenciada na preparação para o cuidado de um filho prematuro.

Para garantir o rigor da pesquisa qualitativa^(14)^, pautada na credibilidade, foi realizada a verificação durante as entrevistas por meio de sínteses orais ou repetições, a fim de assegurar que as interpretações do pesquisador fossem consistentes com as falas do participante. Também foi realizada a triangulação com a assessora durante a análise, para revisar e validar os achados emergentes e identificar possíveis situações que pudessem influenciar as interpretações. Adicionalmente, as entrevistas e os resultados foram devolvidos aos participantes. Quanto à confirmabilidade, foi mantido um arquivo que incluía documentação detalhada do contexto do estudo, participantes, características, procedimentos e processo de análise dos dados, incluindo áudios, transcrições das entrevistas, memorandos, matrizes e diagramas. Os processos e detalhes do estudo foram devidamente registrados e organizados em um arquivo, o que permitirá que outros pesquisadores e profissionais julguem e apliquem seus resultados em contextos e populações semelhantes ao estudado, permitindo assim sua transferibilidade.

### Segunda fase

#### Tipo de estudo

Estudo-piloto randomizado (EPR) com delineamento paralelo e não mascarado^(15)^. Este tipo de estudo envolve todos os processos de um ensaio clínico, mas com um tamanho de amostra menor, e se concentra em estabelecer a viabilidade, aceitabilidade e efeito preliminar da intervenção^(16)^.

#### Período

O período total de recrutamento e acompanhamento foi de 6 meses, terminando em julho de 2022.

#### População e critérios de seleção

Mães de bebês prematuros com idade gestacional igual ou inferior a 34 semanas, internados em Unidade de Terapia Intensiva Neonatal (UTIN), por pelo menos 48 horas após a admissão.

#### Critérios de exclusão

Mães com experiência anterior no cuidado de crianças prematuras e/ou crianças com malformações congênitas.

#### Amostra

Nos EPRs os cálculos formais de tamanho amostral não costumam ser realizados, porém, há recomendações quanto a um número mínimo de participantes, estimado entre 10 e 12 por grupo^(17)^. Assim, participaram 28 mães, sendo 14 em cada grupo.

#### Randomização

Um processo simples de randomização foi realizado no EPIDAT 4.2. A sequência gerada era completamente desconhecida para a pesquisadora, sendo utilizados envelopes opacos lacrados. Todas as sessões de intervenção foram conduzidas pela pesquisadora principal. Um grupo recebeu a intervenção do estudo e o outro grupo recebeu a preparação usual para a alta. As variáveis de resposta foram medidas em três momentos: antes da intervenção (linha basal); uma medição próxima à alta (um dia antes da alta ou no dia da alta) e uma semana após a alta (dias 7-9 após a alta). A variável de resposta principal foi a competência em cuidar de um bebê prematuro em casa. As variáveis de resposta secundárias incluíram ganho de peso, tempo total de internação hospitalar, amamentação exclusiva e outras.

#### Coleta de dados

Para a mensuração das variáveis de interesse, foram utilizados instrumentos *ad hoc* e para a variável resposta principal foi utilizada a escala CUIDAR Ma-Pre, que foi adaptada da escala CUIDAR, elaborada e validada na Colômbia para mensurar a competência de cuidado, entendida como a capacidade da pessoa ou de seu cuidador de realizar seu trabalho de cuidado em casa^(18)^. Embora a escala tenha sido aplicada em diversos contextos, ela não havia sido utilizada em mães de crianças prematuras, sendo necessária sua adaptação, resultando na versão CUIDAR Ma-Pre. Para esta nova versão, foi realizado um processo de adaptação semântica e um julgamento de especialistas, no qual foi alcançado um Índice de Validade de Conteúdo (IVC) = 0,816. A concordância entre os juízes foi moderada, com um índice Kappa de Fleiss de 0,51. Após a aplicação empírica do instrumento a 207 mães, foi realizada uma análise fatorial exploratória na qual a adequação da amostra foi estabelecida por uma estatística de Kaiser-Mayer- Olkin de 0,859 e teste de esfericidade de Bartlett de 2953,9 (p = 0,00). Após o levantamento de vários cenários, um modelo de 7 dimensões e 33 itens explicou 57,9% da variância, com um alfa de Cronbach de 0,852. Os testes de bondade do ajuste permitiram estabelecer por significância estatística de χ ^2^ = 0,01, CFI=0,92, BIC ≥ 10 e RMSEA = 0,05 que o modelo teve um bom ajuste^(19)^. As dimensões do instrumento CUIDAR Ma-Pre são: atua, enfrenta, vínculo, suporte social, conhecimento geral, singularidade e conhecimento específico.

#### Processamento e análise de dados

Os dados foram armazenados e analisados usando o *software* SPSS versão 27.0. Medidas de tendência central, dispersão e posição foram calculadas para variáveis quantitativas, e frequências absolutas e relativas foram usadas para variáveis qualitativas. Para comparar os grupos nas suas condições basais, em relação às variáveis de interesse, foram realizados testes de hipóteses para grupos independentes e o teste t de Student ou Qui-quadrado, de acordo com a distribuição dos dados e a natureza das variáveis. Em todos os casos, assumiu-se significância estatística com valor de p inferior a 0,05, com um nível de confiança de 95%. O teste t de Student foi usado para comparar as pontuações de competência do cuidador e analisar diferenças segundo os momentos de medição. Um modelo ANOVA foi usado para determinar se quaisquer variáveis de interesse teórico ou estatístico estavam envolvidas.

#### Aspectos éticos

O estudo foi aprovado pelo Comitê de Ética em Pesquisa da Faculdade de Enfermagem da Universidade de Antioquia (CEI-FE 2020-02) e pela instituição de saúde onde o estudo foi realizado (CI-HGM 10-0709).

## Resultados da fase qualitativa

Participaram doze mães e quatro pais: cinco com formação universitária, quatro com formação técnica, cinco com ensino médio e dois com ensino fundamental. Cinco eram pais de gêmeos. O peso mínimo foi de 625 gramas e o máximo, de 2.135 gramas. A idade gestacional mínima foi de 26 semanas e a máxima, de 34 semanas.

Os resultados indicam que a preparação para a alta e o fortalecimento da competência dos pais para cuidar do bebê em casa são processos complexos e dinâmicos. Emergiram quatro categorias: 1) Enfrentando o parto prematuro, 2) Vivenciando a experiência cotidiana da prematuridade na Unidade Neonatal, 3) Empoderando-se para o cuidado do bebê prematuro e 4) Cuidando do filho prematuro em casa.

## Enfrentando o parto prematuro

Indica que o parto prematuro é uma situação desconhecida para os pais, que precisam enfrentar uma realidade nova e complexa, diferente da que esperavam. O ideal do parto é ter o filho, levá-lo para casa e assumir os desafios da parentalidade em seu ambiente, mas o parto prematuro é uma experiência diferente. Esta categoria é composta pelas subcategorias: “compreendendo as causas do parto prematuro vivendo em um cenário inimaginável”, “vivendo entre o medo e a esperança” e “conhecendo o filho prematuro”. Alguns depoimentos ilustram isso: *tudo foi muito complexo e surpreendente; eu nunca imaginei que meu filho seria prematuro* (P3). *Tudo foi tão difícil, porque você imagina ter seu filho, ir para casa e tudo vai ficar bem, mas para nós foi diferente* (P5). Ao enfrentar um parto prematuro, é essencial ter apoio e informações que facilitem esse processo.

## Vivenciando o cotidiano da prematuridade na Unidade Neonatal

Composto pelas subcategorias: “vivendo altos e baixos”, “interagindo com o filho prematuro”, “cangureando o filho prematuro”, “amamentando e cangureando na unidade”. Representa as experiências cotidianas e comuns no processo de cuidado dos filhos. Assumir a maternidade e a paternidade neste contexto envolve incerteza, medo, dor, além de uma grande necessidade de apoio, orientação e informação: *hoje estamos bem, amanhã estamos mal, com eles nunca se sabe, todo bebê prematuro é assim, e a pessoa fica com medo porque o curso deles é muito incerto* (P3). No cotidiano da prematuridade, os pais iniciam a interação e o contato com seus filhos de forma limitada, o contato pele a pele pelo do método canguru se expressa como uma forma específica de cuidado e interação: *Eu o coloquei no meu peito e foi como se estivéssemos conectando as nossas almas, como se estivéssemos nos reencontrando* (P5).

Esta categoria reflete a complexidade das experiências dos pais na unidade neonatal. Este espaço inicialmente desconhecido torna-se realidade para eles.

## Empoderando-se para o cuidado do bebê prematuro

É uma fase de apropriação e empoderamento para o cuidado, que facilita a transição para o cuidado domiciliar e fortalece a competência para cuidar do filho prematuro. Os pais não apenas aprenderam sobre o cuidado, mas também superaram medos e deixaram de ser receptores para assumir a responsabilidade pelo papel: *Eu queria fazer tudo, porque eles são meus filhos, então eu queria aprender* (P5); *para frente porque não podemos voltar atrás, é nos empoderar e aceitar a situação* (P11).

O empoderamento surge da experiência como uma forma de enfrentar as dificuldades vivenciadas durante o processo e se fortalecer para assumir o cuidado dos filhos. É composto pelas subcategorias: “aprendendo a cuidar de uma criança prematura”, “reconhecendo as minhas habilidades” e “vivenciando a preparação para a alta”.

## Cuidando do filho prematuro em casa

Composto pelas subcategorias: “momento da alta”, “chegada em casa”, “recebendo apoio” e “acompanhamento após a alta”. O cuidado domiciliar confronta os pais com o cuidado independente de seus filhos, longe do ambiente hospitalar. A transição para casa é um novo começo: a alta da unidade os confronta com novas emoções e uma realidade desconhecida. Embora os pais tenham se preparado para esse momento, a complexidade da experiência se estende ao lar e os confronta com uma mistura de sentimentos e o medo de possíveis complicações: *Eu tinha medo de que, de repente, em casa, ele ficasse doente, que não conseguisse respirar* (P9).

A chegada em casa confronta os pais com a maternidade e a paternidade reais e genuínas, não condicionadas pelo contexto e pelos protocolos da unidade neonatal: *uma vez em casa, a pessoa se sente mais livre para ser mãe* (P1). O apoio familiar é essencial, pois bebês prematuros têm altas demandas de cuidados. O monitoramento do Método Canguru permite a conexão com profissionais de saúde e o apoio para o cuidado domiciliar.

## O empoderamento como categoria central na preparação para o cuidado de um filho prematuro

O empoderamento foi fundamental para o fortalecimento da competência para o cuidado e a transição para o lar. Refere-se às condições e eventos que permitiram aos participantes reconhecerem suas habilidades, acreditar em si mesmos, superar seus medos e se envolver ativamente no cuidado de seus filhos na unidade neonatal. Também mobilizou força e determinação para o cuidado. Uma maior participação no processo de preparação para o cuidado levou a um maior empoderamento. As informações recebidas, o apoio, os altos e baixos vivenciados e a relação de confiança com a equipe de enfermagem contribuem para o empoderamento e definem uma perspectiva individual no processo.

### Integração do método misto: Desenho da Intervenção. Ações para o Cuidado Neonatal Empoderado - ACUNE

Os resultados do componente qualitativo foram conectados à fase quantitativa, orientando o desenho da intervenção, sua abordagem teórica, sua estrutura e os procedimentos de desenho experimental. A Figura 2 mostra a integração realizada com base no desenho da intervenção.

**Figura 2-:** Conexão dos achados qualitativos com a intervenção e a fase quantitativa

**Achados qualitativos**	**Conexão com o desenho da intervenção e procedimentos da fase experimental**
**Categoria principal: Empoderamento para o cuidado do filho prematuro**	Base teórica da intervenção: empoderamento para o cuidado do filho prematuro
**Categoria: Enfrentando o parto prematuro** . Expressa a complexidade da situação no momento inicial; tudo é desconhecido, e os pais precisam de apoio e orientação para se posicionar na situação e no contexto. **Categoria: Vivenciando o cotidiano da prematuridade na Unidade Neonatal.** Tem a ver com a adaptação a um ambiente desconhecido, no qual é necessária a ajuda da equipe de enfermagem para se envolver no cuidado dos filhos e confiar em suas capacidades. **Categoria: Cuidando do filho prematuro em casa.** Parte da necessidade de mobilização e transição para uma nova experiência no processo; novos medos emergem diante do desconhecido e diante de suas capacidades de cuidado.	**Sessão 1.** Primeira semana de hospitalização: *Reconhecendo a prematuridade e o ambiente da UTIN* . O conteúdo e a forma de entrega são guiados pelas experiências dos pais. Os instrumentos são aplicados e a intervenção é aplicada após o primeiro dia, pois, de acordo com a experiência dos pais, não é possível processar a intervenção no primeiro dia. **Sessão 2.** Segunda a terceira semana de internação (após a primeira sessão e antes da terceira sessão): *Compreendendo as especificidades dos cuidados com um filho prematuro* . Inclui conteúdo sobre cuidados com um filho prematuro e contato direto com o método canguru, alimentação, higiene nasal e outras atividades práticas. Baseia-se no apoio e na promoção da segurança e da confiança. **Sessão 3** : Dentro dos 2 dias prévios à alta: *Preparando-se para ir para casa.* Inclui informações e apoio para facilitar a transição. A família é envolvida e recebe orientação sobre recursos pessoais e sociais para o empoderamento. Inclui aspectos relacionados a sinais de alerta, processos administrativos e assistência com o programa de acompanhamento.
**Categoria principal: Empoderamento para o cuidado**	Elaboração de cartilha, manual de intervenção, relacionamento empático, apoio em atividades práticas de cuidado, incluindo atividades de demonstração e simulação. Definição dos momentos adequados para a aplicação dos instrumentos e questionários, bem como para a entrega da intervenção

#### Fundamento teórico da intervenção

O empoderamento é um aspecto central para fortalecer a competência para o cuidado do filho prematuro, pois favorece a preparação para a alta e a transição para o domicílio. A intervenção ACUNE é orientada pela teoria do empoderamento para a saúde^(20)^, que assume o ser humano integralmente com seu ambiente na vida diária e em sua experiência de saúde; caracterizada por padrões de auto-organização, diversidade e mudança inovadora; além disso, reconhece valores e pontos de vista individuais sobre a saúde. A teoria identifica que o empoderamento emerge de recursos pessoais e sociocontextuais^(20)^. Os recursos pessoais refletem características únicas das pessoas e os recursos sociocontextuais incluem o apoio social e de profissionais da saúde. Nessa perspectiva, reafirma-se que o empoderamento é um processo dinâmico de saúde, que enfatiza a possibilidade de participar intencionalmente de um processo de mudança de si mesmo e do ambiente, reconhecendo padrões e comprometendo-se com recursos para o bem-estar. O empoderamento facilita a participação consciente nas decisões do processo de cuidado à saúde^(20-21)^. O empoderamento descrito pelos pais coincide com a perspectiva teórica.

#### Descrição da intervenção ACUNE

A especificação e a estrutura da intervenção foram baseadas na proposta de Sidane e Braden^(22)^. A intervenção ACUNE visa fortalecer a competência para o cuidado dos filhos prematuros em casa, por meio de uma intervenção de empoderamento durante a preparação para a alta da unidade neonatal. Inclui três componentes: cognitivo, social e comportamental.

O componente cognitivo visa fortalecer os recursos pessoais para o empoderamento em saúde e considera os conhecimentos e as habilidades que as mães devem adquirir para cuidar de seus filhos prematuros, o que constitui um recurso pessoal para o empoderamento. O componente social busca promover o reconhecimento de recursos sociais contextuais para o empoderamento. Inclui atividades que visam facilitar o reconhecimento de redes de apoio e de serviços de atenção à saúde. Por fim, o componente comportamental visa promover a participação consciente no processo de preparação para a alta e no fortalecimento da competência para o cuidado do filho prematuro em casa.

A intervenção é realizada presencialmente, com atividades de simulação ou de cuidado direto, dependendo das condições clínicas das crianças. Durante as sessões, são oferecidas oportunidades para perguntas e a expressão de emoções e necessidades em relação à situação. Entre as sessões, são realizadas visitas para abordar as dúvidas das mães e incentivá-las a participar das atividades educativas da instituição de saúde. A intervenção também inclui uma sessão de acompanhamento no dia da admissão no programa canguru para facilitar a transição para o novo ambiente de cuidado.

Inclui uma cartilha educativa como ferramenta informativa e orientadora. Cada seção é acompanhada por espaços para registro das mães, permitindo que descrevam suas emoções, reflexões, dúvidas ou necessidades. Inclui também cápsulas motivacionais e o reconhecimento de seus recursos pessoais e sociais para o empoderamento. O componente de transição inclui uma lista de verificação que a mãe preenche, avaliando seus conhecimentos e habilidades para o cuidado e incentivando-a a solicitar apoio com base em sua própria identificação de necessidades e recursos. A intervenção e os materiais foram avaliados por três especialistas na área, com formação de doutorado em enfermagem e experiência em desenho de intervenções, encontrando consenso sobre sua coerência, adequação e relevância. Suas sugestões foram levadas em consideração antes do início do processo de avaliação.

## Resultados da fase quantitativa

Durante o período de recrutamento houve 146 internações, das quais 32 (21,9%) preencheram os critérios de inclusão; a taxa de recrutamento foi de 87,5%. Das mães do grupo de intervenção, quatro não concluíram a sessão de acompanhamento por motivos administrativos, sugerindo ajustes no protocolo para minimizar a rotatividade. A Figura 3 mostra o fluxograma dos participantes do estudo piloto e a Tabela 1 descreve as variáveis sociodemográficas por grupo de estudo, o que demonstra a comparabilidade dos grupos.

**Figura 3-:** Fluxograma dos participantes do estudo

Nota: Adaptado^10^.

*ACUNE = Ações para o Cuidado Neonatal Empoderado

**Tabela 1-:** Variáveis sociodemográficas por grupos de estudo. Medellín, Antioquia, Colômbia, 2021

Variável	Grupo Controle n*=14	Grupo Intervenção n*=14	Sig ^†^
Idade da mãe	25,14 ± *7,44*	26,93 ± 5,78	0,485 ^‡^
Peso em gramas ao nascer	1327,64 ± 279,31	1695,64 ± 213,60	0,001 ^‡^
Idade gestacional ao nascer	31,21 ± 1,42	32,12 ± 1,16	0,076 ^‡^
Número de filhos	2,93 ± 2,303	2,14 ± 1,027	0,701 ^§^
Competência para o cuidado	81,43 ± *15,37*	80,71 ±16,85	0,908 ^‡^
**Estrato socioeconômico**			
Estrato 1	4	6	0,301 ^||^
Estrato 2	2	3	
Estrato 3	0	1	
Estrato 4	0	1	
Não sabe	8	3	
**Sistema de saúde da previdência social**	8	7	
Subsidiado	0	3	0,177 ^||^
Contributivo	6	4	
Vinculado			
**Nível educacional**	4	0	
Ensino fundamental incompleto	2	1	0,179 ^||^
Ensino fundamental completo	2	2	
Ensino médio incompleto	6	8	
Ensino médio completo	0	2	
Técnico	0	1	
Universitário			
**Área de residência**			
Urbana	10	11	0,663 ^||^
Rural	4	3	
**Nacionalidade**			
Colombiana	10	9	0,686 ^||^
Venezuelana	4	5	
**Estado civil**			
Solteira	4	0	0,094 ^||^
Casada	1	1	
União estável	9	13	
**Primeiro filho**			
Sim	7	9	0,445 ^||^
Não	7	5	
**Multípara**			
Sim	2	3	0,622 ^||^
Não	12	11	
**Peso para a idade gestacional**			
Abaixo do peso	4	2	0,648 ^||^
Peso adequado	10	12	
**Apoio do pai**	1		
Sim	1	14	0,067 ^||^
Não	3	0	

*n = Amostra; ^†^Sig = Significância estatística; ^‡^t = Teste t de Student; ^§^U = Teste U de Mann-Whitney’s; ^||^Chi^2^ = Teste de Qui-quadrado

Em relação ao efeito preliminar, na comparação entre os grupos, aplicando-se o teste t de Student para amostras independentes, constatou-se que a competência para o cuidado melhorou em ambos, porém a diferença não foi significativa. Medição basal: Controle (n=14) 81,43 ± 15,37 vs Intervenção (n=14) 80,71 ± 16,85 p=0,908; medição na alta: Controle (n=13) 117,08 ± 6,103 vs Intervenção (n=14) 121,14 ± 7,156 p=0,126 e medição uma semana após a alta: Controle (n=7) 112,56 ± 6,241 vs Intervenção (n=9) 118,67 ± 9,421 p=0,163.

Na comparação intragrupo, utilizando o teste t de Student para amostras relacionadas, identificou-se que o nível de competência no momento da alta hospitalar aumentou em relação à medida basal, encontrando diferença estatisticamente significativa a favor da intervenção, sendo então calculado o “d” de Cohen para determinar o tamanho do efeito: Controle (n=13): basal 82,15 ± 15,74 vs. alta 117,08 ± 6,10 p=0,000, d de Cohen 12,72; Intervenção (n=14) basal 80,71 ± 16,85 vs. alta 121,14 ± 7,15 p=0,000, d 18,55.

Essa mesma tendência foi identificada na medição uma semana após a alta em relação à medição basal: Controle (n=7): basal 86,71 ± 17,14 vs uma semana após a alta 112,57 ± 6,24 p=0,004, d 15,302; Intervenção (n=9): basal 81,22 ± 16,10 vs uma semana após a alta 118,67 ± 9,421 p=0,000, d 16,853.

No caso da diferença entre o momento da alta e uma semana depois, foi encontrado o seguinte: Controle (n=7) alta 117,43 ± 7,829 e uma semana após a alta 112,57 ± 6,241 p=0,192 vs Intervenção (n=9) alta 121,33 ± 7,365 e uma semana após a alta 121,14 ± 7,156 p=0,335; mas a diferença não foi estatisticamente significativa.

Em relação ao efeito da intervenção ACUNE em variáveis secundárias importantes, diferenças estatisticamente significativas foram encontradas apenas entre os grupos de estudo nos dias de permanência na atenção primária: Controle n=13: 11,38 ± 10,054 vs Intervenção n=13: 4,14 ± 2,656 p=0,025).

Foi explorada a potencial interação de variáveis que poderiam influenciar a diferença nas pontuações. Uma ANOVA de medidas repetidas foi aplicada às três medidas de competência do cuidador e não foram encontradas diferenças estatisticamente significativas que indicassem que qualquer uma delas pudesse influenciar o resultado da competência de cuidado.

Por fim, identificou-se que a intervenção ACUNE apresentou aceitabilidade adequada. Todas as mães indicaram que consideraram a intervenção importante, útil, clara e apropriada. Em relação à estrutura da intervenção, 50% das mães consideraram a duração de cada sessão curta e 57,1% consideraram que as sessões eram poucas. A avaliação da aceitabilidade também incluiu perguntas sobre a cartilha, sua utilidade, adequação, apresentação e clareza. Todas as mães a classificaram como “útil” e “muito útil”. No que diz respeito à apresentação, clareza e conteúdo da cartilha, foram considerados como excelentes pela maior parte das mães.

A Figura 4 mostra a conexão de dados por meio de exposição conjunta, em que os achados qualitativos buscam explicar e complementar os achados quantitativos estatisticamente significativos obtidos na segunda fase, para gerar meta-inferências a partir dos dados.

**Figura 4-:** Apresentação conjunta de dados e meta-inferências

**Resultados quantitativos** **Competência para o cuidado (Intragrupo)**	**Resultados qualitativos** **Empoderando-se para o cuidado: depoimentos**	**Meta-inferências**
**Grupo Controle (n=13)** Medição basal 82,15 ±15,74 Na alta 117,08 ± 6,10 p=0,000 **Grupo Intervenção (n=14)** Medição basal 80,71 ±16,85 Na alta 121,14 ± 7,15 p=0,000	*[…] Eu acredito que a pessoa tem que se empoderar, esses são meus filhos e é isso que eu vou ter que fazer com eles e eu tenho que aprender isso sem medo […]* (P5) *.* *[…] Seguir em frente porque não dá para voltar atrás, é se empoderar e aceitar a situação como ela vem, quando você entende é que você aprende, algumas enfermeiras me disseram que eu conseguiria, que eu deveria confiar em mim mesma, que se outras mães conseguiram, eu também conseguiria e foi isso que mais me ajudou […]* (P11) *.*	A intervenção, cujo foco central foi o empoderamento, demonstrou melhorias preliminares na competência de cuidado, consistentes com as experiências dos pais, que relataram que o empoderamento foi essencial para aprender a cuidar de seus filhos. Os pais precisam de mais do que conhecimento; eles precisam de apoio para superar seus medos em relação à fragilidade de seus filhos e se conectar com o cuidado na unidade neonatal. Uma intervenção de enfermagem empoderadora tem um efeito positivo na competência para o cuidado.
**Grupo Controle (n=7)** Medição basal 86,71 ± 17,14 Uma semana após a alta 112,57 ± 6,24 p=0,004 **Grupo Intervenção (n=9)** Basal 81,22 ± 16,10 Uma semana após a alta 118,67 ± 9,421 p=0,000	*[…] Eu disse que se eles vão me deixar levá-la para casa é porque ela está bem e eles veem que ela consegue respirar sozinha e porque eu vou poder cuidar dela […]* (P8). *[…] Finalmente vai acontecer o que estávamos esperando: estarmos preparados para estar em nossa casa […]* (P5) *.* *[…] já estavamos vindo para casa, mas lá estávamos nós com as enfermeiras e era* só apertar um botão que resolv *iamos o problema, aqui em casa se assusta muito mais […]* (P9).	A intervenção foi elaborada para promover o empoderamento após a alta hospitalar, e os resultados do estudo experimental demonstraram um efeito favorável da intervenção nesse sentido. Embora os resultados sejam preliminares, eles sugerem a conveniência de incluir essa abordagem teórica nos processos de preparação para a alta. Em contrapartida, os resultados mostram que o nível de competência na alta é maior do que o nível registrado uma semana após a alta, indicando que a primeira semana em casa é crítica para os pais, que enfrentam a realidade do cuidado independente, aspecto relatado nas entrevistas.
**Dias de permanência em cuidados básicos** Controle (n=13): 11,38 ± 10,054 Intervenção (n=13): 4,14 ± 2,656 p=0,025 Em termos de aceitabilidade, 50% das mães perceberam que o tempo de cada sessão foi curto e 57,1% que as sessões foram poucas.	*[…] Eu já queria voltar para casa, eu já me sentia preparada, eu já tinha feito, eu fiz lá na unidade, eu já queria ir embora rápido, mas porque eu já sabia cuidar do meu filho e eu me sentia segura […]* (P5). *[…] Eu sempre dizia: ‘Eu vou fazer isso!’ E eles me perguntavam: ‘Você é capaz?’ Eu dizia que não, mas que eu tinha que aprender, então, quando eu o levar, o que eu vou fazer? Eu não vou permitir que eles venham. Eu quero aprender aqui, aprender com vocês, e quanto mais vocês me ensinarem, melhor […]* (P1).	Resultados preliminares mostram que a intervenção reduz o número de dias passados na atenção primária, que é o ambiente de atendimento pré-alta. Mães empoderadas sentem-se confiantes e demonstram sua competência ao avaliar esse critério de alta. Considerando o empoderamento como um elemento diferenciador, além dos materiais e do método de parto, as mães esperam mais sessões e tempo sob essa perspectiva. Com base em sua experiência, maior apoio direto gera maior confiança no cuidado.

## Discussão

Os achados qualitativos ratificam a complexidade e a multidimensionalidade de ter um filho prematuro^(3)^. A preparação para a alta não é um processo isolado; ela está atrelada às experiências que envolvem o nascimento, a hospitalização na unidade neonatal e o cuidado com a criança em casa. As participantes não definem um início e um fim em termos de preparação para o cuidado com seus filhos; esse preparo se reafirma como um processo contínuo que se inicia na admissão na unidade neonatal e não termina com a alta hospitalar^(3,23)^.

A experiência dos participantes começa com o nascimento prematuro e as experiências que os confrontam com uma realidade inesperada. Posteriormente, eles se familiarizam com a vida diária da unidade neonatal, onde criam vínculos com seus filhos, se fortalecem e se preparam para o cuidado domiciliar. Finalmente, eles retornam para casa e enfrentam uma realidade em que programas de cuidado, apoio e acompanhamento desempenham um papel fundamental na experiência. Esses achados são consistentes com outros estudos qualitativos que exploraram as experiências de pais de crianças prematuras. Eles descobriram que, como neste estudo, a hospitalização e a transição para casa marcam um percurso complexo e dinâmico em direção ao cuidado domiciliar competente^(23-25)^. Essas descobertas foram integradas ao desenho da intervenção, sua implementação e aos procedimentos do próprio estudo experimental. Por meio da integração de métodos mistos, os resultados foram considerados consistentes e confirmados. Semelhante às experiências dos pais, onde eles expressaram que quando a equipe de enfermagem os ajudou a se sentirem capazes e fortalecidos, eles foram capazes de cuidar melhor de seus filhos, a intervenção intencional e orientada para o empoderamento alcançou um efeito preliminar positivo em favor da intervenção.

O cotidiano da unidade neonatal é marcado pela necessidade de informações sobre a saúde das crianças e a dinâmica de cuidado específica do contexto, fatores já identificados em outros estudos^(22-24)^. Isso norteia a relevância da inclusão de informações para familiarização com a unidade neonatal, visto que ela é desconhecida e estressante^(24-26)^. Esse aspecto foi fundamental para a intervenção e constituiu o eixo central da primeira sessão, criando uma perspectiva de adequação na abordagem inicial do processo educativo.

Com base na experiência dos participantes, o empoderamento é fundamental para fortalecer a competência no cuidado do filho em casa. Ele surge da assunção do papel parental e do sentimento de responsabilidade pelo cuidado dos filhos. Esses eventos mobilizam força e determinação para se envolver no cuidado e superar medos. Informações e apoio da equipe de enfermagem e das famílias contribuem para o empoderamento, que se inicia na unidade neonatal e se fortalece na alta. A necessidade de empoderamento esteve presente em outros estudos, que reafirmam que o apoio à participação ativa no cuidado dos filhos dentro da unidade é um mediador do empoderamento, contribuindo para a segurança e a confiança para a transição^(27-28)^. A intervenção ACUNE, ao focar no empoderamento, considera a experiência dos pais e os toma como ponto de partida, demonstrando que essa abordagem gera resultados positivos para a experiência e a capacidade de cuidado dos pais.

O empoderamento tem sido abordado na educação em saúde como parte da tendência em direção ao cuidado e atenção participativos^(29-30)^. Isso reafirma a importância do empoderamento nos processos de preparação dos pais para a alta da unidade neonatal e favorece a integração da teoria do empoderamento na intervenção ACUNE.

De acordo com a teoria, o empoderamento em saúde é um processo relacional que surge do reconhecimento do indivíduo, de seus recursos pessoais e do contexto social^(19-20)^. Este princípio é consistente com as experiências dos participantes e constitui um aspecto central da intervenção ACUNE, cuja avaliação preliminar indica que é viável, aceitável e pode gerar um efeito positivo na competência para cuidar de um filho prematuro. A intervenção demonstrou ser viável com base em recursos físicos e humanos. A simplicidade de sua estrutura torna possível sua utilização com as mães conforme planejado. Ao longo do estudo, a mensuração das variáveis de interesse foi considerada viável e os instrumentos foram aplicados sem dificuldade.

Ao analisar os resultados da competência em cuidados domiciliares entre os grupos, não foram encontradas diferenças estatisticamente significativas, mas identificou-se que a pontuação da competência aumentou em ambos os grupos, sendo maior no grupo de intervenção. As mães de ambos os grupos receberam a intervenção usual, mas as mães do grupo de intervenção receberam adicionalmente a intervenção ACUNE, que, diferentemente da intervenção usual, possui uma estrutura definida e é articulada dentro de um quadro teórico disciplinar de empoderamento.

Ao avaliar o efeito intragrupo, foi possível evidenciar que existem mudanças favoráveis em ambos os grupos, de acordo com os momentos de medição, e nesta análise foram identificadas diferenças estatisticamente significativas. Este resultado preliminar indica que a intervenção poderia melhorar a competência das mães para cuidar dos seus filhos prematuros em casa, o que sugere que é pertinente realizar o estudo randomizado em maior escala.

Ao comparar os resultados com outros estudos que buscaram estabelecer o efeito da abordagem de empoderamento, é importante reconhecer que as evidências mostram que ela tem efeitos favoráveis. Um estudo recente realizado com 80 mães, 40 em cada grupo, constatou que uma intervenção de aconselhamento voltada para mães de crianças prematuras apresentou diferença significativa na pontuação média de autoestima 9,52 ± 3,22 e 16,75 ± 6,39 (p < 0,001) e estresse 154,65 ± 32,15 e 61 ± 10,98 (p < 0,001). Dentro do estresse, a redução mais significativa foi identificada na dimensão de aparência e comportamento do bebê (d de Cohen = 3,146)^(29)^. Este achado reafirma a consistência da intervenção ACUNE com as evidências, pois foram incluídas informações relacionadas a este tema, com base nos achados da primeira fase, onde pelas experiências dos pais, identificou-se que o aparecimento inicial do filho prematuro era chocante e gerava medo durante o cuidado e contato direto com seu filho.

Um aspecto marcante é que, embora a aferição na alta e uma semana após a alta no grupo intervenção seja maior que no grupo controle, identificou-se que há uma diminuição da pontuação após a alta, o que tem a ver com o fato de que a primeira semana de transição para casa é crítica, e os pais enfrentam novos desafios que podem questionar sua capacidade e sua confiança pode ser diminuída^(28)^.

Nesta análise, é importante considerar que os resultados referentes ao efeito são preliminares. O pequeno tamanho da amostra proporciona pouco poder estatístico, fator ao qual devemos somar, neste caso particular, o elevado número de perdas. Esses aspectos são de certa forma esperados em estudos piloto, razão pela qual as possibilidades de encontrar diferenças e conduzir outros tipos de análise são limitadas. Portanto, a interpretação dos resultados é cautelosa e não conclusiva quanto ao efeito, embora seja orientadora. Também permite ponderar os aspectos relacionados à viabilidade e aceitabilidade, otimizando tempo e recursos na condução do ensaio clínico randomizado.

A aceitabilidade da intervenção foi bo, e o principal ajuste sugerido após o estudo piloto foi a viabilidade do acompanhamento das mães encaminhadas para outras instituições. Nesse sentido, é necessário garantir e facilitar o acesso das mães para o acompanhamento pós-alta, utilizando meios virtuais para reduzir as perdas.

Esses resultados reafirmam que o empoderamento em saúde é uma perspectiva teórica que permite aprimorar as habilidades e a confiança das mães no cuidado de seus filhos prematuros. Emerge da experiência dos pais e é confirmada pela avaliação de uma intervenção guiada por essa abordagem, que é socialmente sensível, validada pelas experiências dos pais e confirmada de forma preliminar por um estudo experimental. Isso abre novos caminhos para a confirmação do efeito e confere valor e significado à pesquisa de métodos mistos, que incorpora as vozes das pessoas envolvidas nos fenômenos de interesse da disciplina.

A avaliação preliminar da intervenção ACUNE indica que ela pode ter um efeito positivo na competência para o cuidado de um filho prematuro em casa. Os resultados são preliminares e não conclusivos, mas fornecem orientações quanto à conveniência de conduzir o estudo principal.

Considera-se como limitação o tamanho da amostra do presente estudo-piloto. Embora cálculos formais não sejam utilizados para este tipo de estudo e seu escopo seja indicativo, uma amostra maior poderia fornecer uma representação mais precisa do efeito real. No entanto, reconhece-se, a partir da sua perspetiva mista, como ponto forte, a conceção de uma intervenção que surge a partir das vozes dos pais e a confirmação preliminar do empoderamento como uma abordagem teórica que melhora a competência para o cuidado dos filhos prematuros.

## Conclusão

A experiência de mães e pais no desenvolvimento e fortalecimento de suas competências no cuidado do filho prematuro é complexa e dinâmica. O empoderamento é um aspecto central, promovendo a capacidade de cuidado, a confiança e a transição para o lar. A participação nos cuidados, o apoio familiar, o acompanhamento e a interação com a equipe de enfermagem são essenciais para o empoderamento. O desenho de uma intervenção de enfermagem para fortalecer a competência no cuidado dos filhos prematuros sob a perspectiva do empoderamento possui uma base teórica que permite sua aplicação prática; além disso, há evidências empíricas que sustentam a adequação desse tipo de intervenção. A integração dos resultados qualitativos ao desenho da intervenção favoreceu a sua sensibilidade social e proximidade com a realidade das mães e com a prática profissional de enfermagem.

# Translation: Diseño y evaluación de una intervención para padres de niños prematuros: estudio de métodos mixtos*

**DOI:** 10.1590/1518-8345.7791.4746

**Authors:** Sandra Patricia Osorio-Galeano, Angela María Salazar-Maya

**Affiliations:** 1 Universidad de Antioquia, Facultad de Enfermería, Medellín, Antioquia, Colombia; 2 Universidad CES, Facultad de Enfermería, Medellín, Antioquia, Colombia

**Keywords:** Enfermería Neonatal, Recién Nacido Prematuro, Padres, Unidades de Cuidado Intensivo Neonatal, Empoderamiento, Investigación en Enfermería

## Abstract

diseñar y evaluar una intervención para fortalecer la competencia para el cuidado de hijos prematuros.

estudio de intervención de métodos mixtos, exploratorio y secuencial. En la primera fase se aplicó una teoría fundamentada. Participaron 12 madres y 4 padres. En la segunda, se implementó un estudio piloto aleatorizado con 14 madres en cada grupo. La variable de respuesta principal fue la competencia para el cuidado. Se aplicó la escala CUIDAR Ma-Pre en la medición basal y en dos mediciones de seguimiento.

surgieron cuatro categorías: Afrontando el parto prematuro, Experimentando la cotidianidad de la prematurez en la Unidad Neonatal, Empoderándose para el cuidado del hijo prematuro y Cuidando al hijo prematuro en el hogar. Empoderándose para el cuidado del hijo prematuro fue la categoría central. Estos resultados se integraron en el diseño de la intervención. En la evaluación se encontraron diferencias a favor de la intervención. La diferencia no fue estadísticamente significativa entre grupos, pero si lo fue intragrupo (Control n=13 basal 82,15; al alta 117,08 p=0,000 vs Intervención n=14 basal 80,71; al alta 121,14 p=0,000)

el empoderamiento fue esencial en la experiencia; una intervención con este enfoque es relevante y su evaluación preliminar sugiere que puede mejorar la competencia para el cuidado. Es pertinente un estudio a mayor escala. ClinicalTrials.gov. Identificador:NCT05005988.

## Destacados

**(1)** El empoderamiento es central en la experiencia de cuidado de los hijos prematuros. **(2)** El diseño de una intervención desde la experiencia de los padres es pertinente. **(3)** El enfoque teórico de empoderamiento es aplicable a las intervenciones educativas. **(4)** Una intervención orientada a padres de niños prematuros es viable y aceptable. **(5)** El empoderamiento podría fortalecer la competencia para el cuidado.

## Introducción

La prematuridad es un tema de gran interés para los profesionales de la salud, puesto que los niños nacidos antes de las 37 semanas de gestación tienen mayores riesgos de enfermar y morir que los niños a término, por lo tanto, su cuidado y atención representan un importante desafío^(1)^. Además, es necesario considerar que el nacimiento prematuro expone a los padres a una experiencia compleja, cargada de culpa, ansiedad, estrés, miedo e incertidumbre^(2-3)^, no solo durante el nacimiento y la hospitalización, sino también durante la transición y el cuidado en elhogar^(2)^.

La continuidad del cuidado después del alta es determinante para el bienestar de los niños prematuros, quienes durante un tiempo requieren cuidados especiales para garantizar su adecuado crecimiento y desarrollo^(4)^. Por esta razón, tanto madres como padres deben adquirir conocimientos y habilidades para el cuidado antes del alta de la unidad neonatal^(4-5)^, pero además es necesario valorar e involucrar aspectos emocionales y sociales que favorecen y facilitan el cuidado en el hogar^(5)^.

Estos aspectos reafirman la necesidad de ampliar la comprensión acerca de las experiencias de los padres en el proceso de preparación para el alta y articularlas en el diseño de intervenciones educativas que permitan fortalecer la competencia para el cuidado de los hijos prematuros. Es así como un aspecto clave del estudio fue el diseño de métodos mixtos. Los hallazgos de una primera fase cualitativa orientaron el diseño de la intervención y su enfoque teórico. Esta fase brindó elementos que ayudaron a establecer la estructura y entrega de la intervención en la realidad de la práctica profesional y permitió diseñar una intervención socialmente sensible y pertinente, considerando las voces de madres y padres que vivieron la experiencia. El objetivo del estudio fue diseñar y evaluar una intervención para fortalecer la competencia para el cuidado de los hijos prematuros.

## Método

### Tipo de estudio

Estudio de intervención de método mixto exploratorio secuencial en el que ambos enfoques tuvieron igual predominancia CUAL → CUAN^(6-7)^. Este tipo de investigación propone una razón de complementariedad desde la perspectiva individual de cada paradigma. El pluralismo dialéctico, sustenta epistemológicamente el estudio, puesto que favorece el dialogo de perspectivas y una investigación cultural, socialmente sensible^(8)^ (Figura 1).

**Figura 1-:** Estructura general del diseño de método mixto

La integración metodológica se dio a partir del enfoque de conexión^(6)^. Los resultados de la primera fase se integraron a la segunda a través del diseño de la intervención. Los resultados cualitativos informaron y orientaron la intervención, su perspectiva teórica, los componentes y estructura operativa y ayudaron a dar forma a la planificación de los procedimientos del diseño experimental del componente cuantitativo. El reporte de la integración durante el análisis fue a través de la narrativa^(6)^.

Considerando la metodología y las fases del estudio, se siguieron los guías: *Consolidated criteria for Reporting Qualitative research* (COREQ)^(9)^, *CONSORT Statement: Extension to Randomised Pilot and Feasibility Trials* ^(10)^ y *Mixed Methods Appraisal Tool* (MMAT)^(11)^ para garantizar la transparencia metodológica y la legitimación de las inferencias derivadas a partir de la validez múltiple, considerando de forma cuidadosa los supuestos que soportan los procesos de investigación de los componentes tanto cualitativos como cuantitativos, con la intención de respaldar los hallazgos del estudio de método mixto^(12)^.

### Primera fase

#### Tipo de estudio

Teoría fundamentada^(13)^.

#### Periodo

La investigación se llevó a cabo entre los meses de mayo de 2020 y febrero de 2021.

#### Población y criterios de selección

Padres y madres de niños prematuros nacidos antes de las 34 semanas de gestación, con egreso hospitalario mayor a 15 días y menor o igual a 6 meses.

#### Criterios de exclusión

Padres o madres cuyos hijos tuvieran malformaciones congénitas o que tuvieran problemas de salud al momento de la entrevista.

#### Participantes y definición de la muestra

Doce madres y cuatro padres. Se realizó un muestreo inicial a conveniencia, y en bola de nieve. Durante el análisis de los datos se siguió un muestreo teórico, que densificó las categorías, hasta alcanzar la saturación teórica que determinó el número de entrevistas a realizar^(13)^.

#### Recolección de datos

Las entrevistas fueron realizadas por la investigadora principal, quien es enfermera docente, con formación postgraduada de maestría y doctorado, con experiencia en investigación cualitativa y quien tiene amplia experiencia profesional y sensibilidad por el cuidado neonatal. Se realizó contacto vía telefónica con los participantes y se les informó acerca de los objetivos, razones e intereses de la investigación y de la investigadora. Una vez aceptaron su participación se agendó la fecha y hora de la entrevista semiestructurada, la cual fue realizada por medio de video llamadas. Ningún invitado se negó a participar. Se aplicó una guía de entrevista cuyas preguntas abarcaban los temas centrales de la preparación para el alta de la unidad neonatal, como por ejemplo vivencias en torno a la forma como recibió la información, experiencias al participar del cuidado del hijo en la unidad y en el cuidado tras el alta, entre otras. A medida que transcurrió el análisis de los datos surgieron nuevas preguntas, que permitieron profundizar en algunos temas a la par con el muestreo teórico, para obtener información sobre el fenómeno y comprender sus dimensiones. Las entrevistas tuvieron una duración que osciló entre 30 y 80 minutos. Cada entrevista fue audio grabada con autorización de los participantes. Se tomaron notas de campo durante cada entrevista para completar la información y enriquecer el análisis.

#### Tratamiento y análisis de los datos

Las entrevistas fueron transcriptas por la investigadora principal dentro de las 48 horas posteriores a su realización. El análisis se llevó a cabo de forma dinámica, no secuencial, mediante comparación constante de los datos^(13)^. Se realizó codificación abierta, con identificación de incidentes y asignación de códigos. Luego se utilizó codificación axial para relacionar categorías con subcategorías y formar unas explicaciones precisas sobre el fenómeno y finalmente, a través de codificación selectiva, se identificó la categoría central, que permitió comprender la trayectoria vivida en la preparación para el cuidado de un hijo prematuro.

Para garantizar el rigor de la investigación cualitativa^(14)^, en función de la credibilidad, se realizó verificación durante las entrevistas mediante síntesis orales o repeticiones, a fin de asegurar que las interpretaciones del investigador fueran consistentes con las declaraciones del participante. También se realizó triangulación con la asesora durante el análisis, para revisar y validar los hallazgos emergentes e identificar posibles situaciones que pudieran influenciar las interpretaciones. Adicionalmente, se realizó devolución de las entrevistas y los resultados a los participantes. En cuanto a la confirmabilidad se mantuvo un archivo que incluyó documentación detallada del contexto del estudio, participantes, características, procedimientos y proceso de análisis de datos, incluyendo los audios, las transcripciones de las entrevistas, memos, matrices y diagramas. Los procesos y los detalles del estudio fueron debidamente registrados y organizados en un archivo, lo que posibilitará a otros investigadores y profesionales juzgar y aplicar sus resultados en contextos y poblaciones similares a la estudiada, permitiendo por lo tanto la transferibilidad de los mismos.

### Segunda fase

#### Tipo de estudio

Estudio piloto aleatorizado (EPA) con diseño paralelo, sin enmascaramiento^(15)^. Este tipo de estudio implica todos los procesos de un ensayo clínico, pero con menor muestra, se centra en establecer factibilidad, aceptabilidad y efecto preliminar de la intervención^(16)^.

#### Periodo

El tiempo total de reclutamiento y seguimiento fue de 6 meses, finalizando julio de 2022.

#### Población y criterios de selección

Madres de niños prematuros con edad gestacional menor o igual a las 34 semanas, hospitalizados en una Unidad de Cuidado Intensivo Neonatal (UCIN), por al menos 48 horas después del ingreso.

#### Criterios de exclusión

Madres con experiencia previa en el cuidado de un hijo prematuro y/o hijos con malformación congénita.

#### Muestra

En los EPA no suelen realizarse cálculos formales para el tamaño de la muestra, sin embargo, existen recomendaciones respecto a un número mínimo de participantes, estimado entre 10 y 12 por grupo^(17)^. Es así, como participaron en total 28 madres, 14 en cada grupo.

#### Aleatorización

Se realizó un proceso de aleatorización simple en EPIDAT 4.2. La secuencia generada fue totalmente ajena a la investigadora y se manejaron sobres opacos sellados. Todas las sesiones de la intervención fueron realizadas por la investigadora principal. Un grupo recibió la intervención en estudio y el otro grupo recibió la preparación habitual para el alta. La medición de variables de respuesta se realizó en tres momentos: antes de la intervención (línea basal); una medición cercana al alta (1 día previo al alta o el día del alta) y una semana después del egreso (días 7-9 después del alta). La variable de respuesta principal fue la competencia para el cuidado del hijo prematuro en el hogar. Como variables de respuesta secundarias se consideraron la ganancia de peso, los días totales de estancia hospitalaria, lactancia materna exclusiva, entre otras.

#### Recolección de datos

Para la medición de las variables de interés se utilizaron instrumentos *ad hoc* y para la variable de respuesta principal se utilizó la escala CUIDAR Ma-Pre, cuya adaptación se realizó a partir de la escala CUIDAR, diseñada y validada en Colombia para medir la competencia para el cuidado, entendida como la capacidad de la persona o su cuidador para ejercer su labor de cuidado en el domicilio^(18)^. Si bien la escala había sido aplicada en diversos contextos, no había sido utilizada en madres de niños prematuros, por lo que fue necesaria su adaptación, que resultó en la versión CUIDAR Ma-Pre. Para esta nueva versión se realizó un proceso de adecuación semántica y un juicio de expertos en el que se alcanzó un índice de Validez de Contenido (IVC)=0,816. La concordancia entre los jueces fue moderada, con un índice Kappa de Fleiss de0,51. Tras la aplicación empírica del instrumento a 207 madres, se realizó un análisis factorial exploratorio en el que se estableció la idoneidad de la muestra por un estadístico Kaiser-Mayer-Olkin de 0,859 y la prueba de esfericidad de Bartlett 2953,9 (p=0,00). Después de plantear diversos escenarios, un modelo de 7 dimensiones y 33 ítems explicó el 57.9% de la varianza, con un alfa de Cronbach de 0,852. Las pruebas de bondad de ajuste permitieron establecer mediante significación estadística de χ^2^=0,01, CFI=0,92, BIC≥10 y RMSEA=0,05 que el modelo tenía un buen ajuste^(19)^. Las dimensiones del instrumento CUIDAR MaPre son: actúa, afronta, vínculo, apoyo social, conocimiento general, unicidad y conocimiento específico.

#### Tratamiento y análisis de datos

Se almacenaron y analizaron con el *software* SPSS versión 27.0. Para variables cuantitativas, se calcularon medidas de tendencia central, dispersión y posición, y para variables cualitativas se realizó descripción mediante frecuencias absolutas y relativas. Para comparar los grupos en sus condiciones basales, con respecto a variables de interés, se realizaron pruebas de hipótesis para grupos independientes y la prueba t de Student o Chi cuadrado según la distribución de los datos y naturaleza de las variables. En todos los casos, se asumió significación estadística con valor de p menor de 0,05 con una confianza del 95%. En la comparación de los puntajes de la competencia del cuidador y en el análisis de las diferencias según momentos de medición, se utilizó la prueba de t de Student. Se exploró un modelo tipo ANOVA para establecer si alguna variable de interés teórico o estadístico intervenía en las mismas.

#### Aspectos éticos

Se contó con aval del Comité de Ética de Investigación de la Facultad de Enfermería de la Universidad de Antioquia N° CEI-FE 2020-02 y de la institución de salud donde se realizó el estudio N° CI-HGM 10-0709.

## Resultados de la fase cualitativa

Participaron 12 madres y 4 padres, 5 con formación universitaria, 4 con nivel técnico, 5 con secundaria y 2 con estudios de primaria. Hubo 5 padres de mellizos o gemelos. El peso mínimo fue de 625 y máximo de 2135 gramos. La edad gestacional mínima fue de 26 y la máxima de 34 semanas.

Los resultados indican que la preparación para el alta y el fortalecimiento de la competencia de los padres para el cuidado de su hijo en el hogar son procesos complejos y dinámicos. Emergieron cuatro categorías: 1) Afrontando el parto prematuro, 2) Experimentando la cotidianidad de la prematurez en la Unidad Neonatal 3) Empoderándose para el cuidado del hijo prematuro y 4) Cuidando en el hogar.

## Afrontando el parto prematuro

Indica que el nacimiento prematuro es una situación desconocida para los padres, quienes deben afrontar una nueva y compleja realidad diferente a la esperada. El ideal del nacimiento es tener a su hijo, llevarlo a su casa y asumir los desafíos de la paternidad en su entorno, pero el parto prematuro es una experiencia diferente. Esta categoría la conforman las subcategorías: “comprendiendo las causas del parto prematuro viviendo un escenario inimaginado”, “viviendo entre el miedo y la esperanza” y “conociendo al hijo prematuro”. Algunos testimonios ilustran esto: *todo fue muy complejo y sorprendente, yo jamás me imaginé que mi hijo iba a ser prematuro* (P3). *Todo era tan difícil, porque uno se imagina tener a su hijo, me voy para mi casa y todo va a estar bien, pero a nosotros nos tocó distinto* (P5). Al afrontar el nacimiento prematuro, es fundamental contar con acompañamiento e información que facilite este proceso.

## Experimentando la cotidianidad de la prematurez en la Unidad Neonatal

Compuesta por las subcategorías: “viviendo altibajos”, “interactuando con el hijo prematuro”, “canguriando al hijo prematuro”, “lactando y canguriando en la unidad”. Representa las experiencias cotidianas y comunes dentro del proceso de atención de sus hijos. Asumir la maternidad y la paternidad en este contexto, implica incertidumbre, miedo, dolor, así como una gran a necesidad de apoyo, acompañamiento e información: *Hoy estamos bien, mañana estamos mal, con ellos no se sabe, todo bebé prematuro es así y uno se llena de miedo porque el transcurso de ellos es muy incierto* (P3). En la cotidianidad de la prematurez los padres inician la interacción y el contacto con sus hijos de forma limitada, el contacto piel a piel mediante el método canguro se expresa como una forma de cuidado e interacción específica: *Yo lo puse en mi pecho y eso fue como si nos estuviéramos compenetrando en nuestras almas, como si nos volviéramos a encontrar* (P5).

Está categoría, da cuenta de la complejidad de las vivencias de los padres en la unidad neonatal. Este espacio inicialmente desconocido, se convierte en su realidad.

## Empoderándose para el cuidado del hijo prematuro

Es una fase de apropiación y empoderamiento para el cuidado, que favorece la transición al hogar y fortalece la competencia para el cuidado del hijo prematuro. Los padres no solo aprendieron acerca del cuidado, sino que también vencieron miedos y pasaron de ser receptores, para apropiarse de rol: *yo quería hacer todo, porque son mis hijos, entonces yo quería aprender* (P5); *para adelante porque para atrás no podemos, es empoderarnos y aceptar la situación* (P11).

El empoderamiento surge desde la experiencia como una forma que les permite enfrentar dificultades vividas en el proceso y fortalecerse para asumir el cuidado de sus hijos. Está conformada por las subcategorías: “aprendiendo a cuidar el niño prematuro”, “reconociendo mis capacidades”, “viviendo la preparación para el alta”.

## Cuidando al hijo prematuro en el hogar

Conformada por las subcategorías: “el momento del alta”, “llegando a casa”, “recibiendo apoyo” y “el seguimiento después del alta”. El cuidado en el hogar enfrenta a los padres al cuidado independiente de sus hijos, lejos del entorno hospitalario. Transitar al hogar es un nuevo comienzo: el alta de la unidad los enfrenta a nuevas emociones y a una realidad que desconocen. Si bien los padres se han preparado para este momento, la complejidad de la experiencia se extiende hasta el hogar y los enfrenta a una mezcla de sentimientos y al temor frente a posibles complicaciones: *me daba temor de que de pronto en la casa se me pusiera mal, que no pudiera respirar* (P9).

La llegada al hogar enfrenta a los padres a una maternidad y una paternidad real, genuina, no condicionada por el contexto y los protocolos de la unidad neonatal: *ya en la casa uno se siente más libre para ser mamá* (P1). El apoyo familiar resulta fundamental, puesto que los niños prematuros tienen altas demandas de cuidado. El seguimiento en el plan canguro permite la conexión con los profesionales de la salud y el apoyo para el cuidado en el hogar.

## El empoderamiento como categoría central en la preparación para el cuidado del hijoprematuro

El empoderamiento fue determinante en fortalecer la competencia para el cuidado y la transición al hogar. Tiene que ver con las condiciones y los eventos que permitieron a los participantes reconocer sus capacidades, creer en ellos, vencer los miedos y vincularse activamente con el cuidado de sus hijos en la unidad neonatal. Además, movilizó fuerza y determinación para el cuidado. A mayor participación en el proceso de preparación para el cuidado, mayor empoderamiento. La información recibida, el apoyo, los altibajos vividos y la relación de confianza con el personal de enfermería aportan al empoderamiento y marcan una perspectiva individual en el proceso.

## Integración del método mixto: Diseño de la intervención. Acciones para un Cuidado Neonatal Empoderado-ACUNE

Los resultados del componente cualitativo se conectaron con la fase cuantitativa al orientar el diseño de la intervención, su enfoque teórico, su estructura, así como los procedimientos del diseño experimental. En la Figura 2, se puede identificar la integración realizada en función del diseño de la intervención.

**Figura 2-:** Conexión de los hallazgos cualitativos con la intervención y la fase cuantitativa

**Hallazgo cualitativo**	**Conexión con el diseño de la intervención y los procedimientos de la fase experimental**
**Categoría central: El empoderamiento para el cuidado del hijo prematuro**	Fundamento teórico de la intervención: el empoderamiento para el cuidado del hijo prematuro
**Categoría: Afrontando el parto prematuro** . Expresa la complejidad de la situación en el momento inicial, todo resulta desconocido, los padres necesitan apoyo y orientaciones para ubicarse en la situación y el contexto. **Categoría: Viviendo la cotidianidad de la prematurez en la unidad neonatal.** Tiene que ver con la adaptación a un entorno desconocido, en el que se requiere ayuda del personal de enfermería para involucrarse en el cuidado de los hijos y confiar en sus capacidades **Categoría: Cuidando el hijo prematuro en el hogar.** Parte de la necesidad de movilizarse y transitar a una nueva experiencia en el proceso, en donde emergen nuevos miedos frente a lo desconocido y frente a su capacidad de cuidado.	**Sesión 1.** Primera semana de hospitalización: *Reconociendo la prematurez y el entorno de la UCIN* . El contenido y la forma de entrega está orientado por las experiencias de los padres. Se realiza la aplicación de instrumentos y la entrega de la intervención después del primer día, porque de acuerdo con la experiencia de los padres el primer día no es posible procesarlo. **Sesión 2.** Segunda a tercera semana de hospitalización (después de la primera sesión y antes de la tercera sesión): *Conociendo las particularidades del cuidado de un hijo prematuro* . Incluye contenidos acerca del cuidado del hijo prematuro y el contacto directo mediante el método canguro, la alimentación, higiene nasal y otras actividades prácticas. Se basa en el acompañamiento y el fomento de la seguridad y la confianza. **Sesión 3** : Dentro de los 2 días previos al alta: *Preparándose para ir a casa.* Incluye la información y acompañamiento para favorecer la transición. Se involucra a la familia y se orienta acerca de los recursos personales y sociales para el empoderamiento. Incluye aspectos relacionados con signos de alarma, procesos administrativos y asistencia a programa de seguimiento.
**Categoría central: El empoderamiento para el cuidado**	Diseño de cartilla, manual del intervencionista, relación empática, apoyo en actividades prácticas para el cuidado, incluyendo actividades de demostración y simulación. Definición de los momentos propicios para aplicación de instrumentos y cuestionarios, así como para la entrega de la intervención

### Fundamento teórico de la intervención

El empoderamiento es un aspecto central para fortalecer la competencia para el cuidado del hijo prematuro, ya que favorece la preparación para el alta, y la transición al hogar. La intervención ACUNE está orientada por la teoría del empoderamiento para la salud^(20)^, que asume al ser humano integralmente con su entorno en la vida diaria y en su experiencia de salud; caracterizado por patrones de auto-organización, diversidad y cambio innovador; además, reconoce los valores y puntos de vista individuales sobre la salud. La teoría identifica que el empoderamiento emerge de recursos personales y socio-contextuales^(20)^. Los personales reflejan características únicas de las personas y los social-contextuales incluyen el apoyo social y del personal de salud. Desde esta perspectiva se reafirma que el empoderamiento es un proceso de salud dinámico, que enfatiza en la posibilidad de participar intencionalmente en un proceso de cambiarse a sí mismo y al entorno, reconocer patrones y comprometerse con los recursos para el bienestar. El empoderamiento facilita la participación consciente en las decisiones del proceso de atención en salud^(20-21)^. El empoderamiento descripto por los padres coincide con la perspectivateórica.

### Descripción de la intervención ACUNE

Para la especificación de la intervención y su estructura se siguió la propuesta de Sidane y Braden^(22)^. La intervención ACUNE tiene como objetivo fortalecer la competencia para el cuidado de los hijos prematuros en el hogar, mediante una intervención de empoderamiento durante la preparación para el alta de la unidad neonatal. Contempla tres componentes: cognitivo, social y comportamental.

El componente cognitivo tiene como meta fortalecer los recursos personales para el empoderamiento de la salud y considera los conocimientos y habilidades que las madres deben adquirir para el cuidado de sus hijos prematuros, que se constituyen en un recurso personal para el empoderamiento. El componente social busca favorecer el reconocimiento de los recursos sociales contextuales para el empoderamiento. Incluye actividades que buscan facilitar el reconocimiento de redes de apoyo y de los servicios de atención en salud. Por último, el componente conductual pretende promover la participación consciente en el proceso de preparación para el alta y en el fortalecimiento de la competencia para el cuidado del hijo prematuro en el hogar.

La entrega de la intervención es cara a cara, se realizan actividades de simulación o cuidado directo según condiciones clínicas de los niños. Durante las sesiones se generan espacios para realizar preguntas, expresar emociones y necesidades frente a la situación. Entre las sesiones se realizan visitas para atender dudas de las madres y se les motiva a participar de las actividades educativas de la institución de salud. La intervención también incluye una sesión de acompañamiento el día del ingreso al “programa canguro”, para favorecer la transición al nuevo espacio de cuidado.

Incluye una cartilla educativa, como herramienta informativa y orientadora. Cada sección se acompaña de espacios de registro para que las madres describan sus emociones, reflexiones, preguntas o necesidades. También incluye capsulas de motivación y reconocimiento de sus recursos personales y sociales para el empoderamiento. El componente que tiene que ver con la transición incluye una lista de chequeo que la madre completa valorando sus conocimientos y habilidades para el cuidado y que la lleva a solicitar apoyo según su propia identificación de necesidades y recursos. La intervención y el material fue valorado por 3 expertas en el área, con formación doctoral en enfermería y experiencia en el diseño de intervenciones, encontrando consenso en términos de su coherencia, idoneidad y relevancia. Sus sugerencias fueron acogidas antes de iniciar el proceso de evaluación.

## Resultados de la fase cuantitativa

Durante el periodo de reclutamiento hubo 146 ingresos, de los cuales 32 (21,9%) cumplieron los criterios de inclusión; el porcentaje de reclutamiento fue de 87,5%. De las madres del grupo de intervención, 4 no completaron la sesión de acompañamiento por situaciones administrativas, lo que sugiere ajustes al protocolo para minimizar las pérdidas. En la Figura 3, se observa el diagrama de flujo de los participantes en el estudio piloto y en la Tabla 1, la descripción de las variables sociodemográficas por grupo de estudio, donde se puede apreciar la comparabilidad de los grupos.

**Figura 3-:** Flujograma de participantes del estudio

Nota: Adaptado10.

*ACUNE = Acciones para un Cuidado Neonatal Empoderado

**Tabla 1-:** Variables sociodemográficas por grupos de estudio. Medellín, Antioquia, Colombia, 2021

Variable	Grupo Control n*=14	Grupo Intervención n*=14	Sig ^†^
Edad de la madre	25,14 ± *7,44*	26,93 ± 5,78	0,485 ^‡^
Peso en gramos alnacimiento	1327,64 ± 279,31	1695,64 ± 213,60	0,001 ^‡^
Edad gestacional alnacimiento	31,21 ± 1,42	32,12 ± 1,16	0,076 ^‡^
Número de hijos	2,93 ± 2,303	2,14 ± 1,027	0,701 ^§^
Competencia para el cuidado	81,43 ± *15,37*	80,71 ±16,85	0,908 ^‡^
**Estrato socioeconómico**			
Estrato 1	4	6	0,301 ^||^
Estrato 2	2	3	
Estrato 3	0	1	
Estrato 4	0	1	
No sabe	8	3	
**Régimen de seguridad social en salud**			
Subsidiado	8	7	0,177 ^||^
Contributivo	0	3	
Vinculado	6	4	
**Nivel educativo**			
Primaria incompleta	4	0	0,179 ^||^
Primaria completa	2	1	
Secundaria incompleta	2	2	
Secundaria completa	6	8	
Técnica	0	2	
Universitaria	0	1	
**Área de residencia**			
Urbana	10	11	0,663 ^||^
Rural	4	3	
**Nacionalidad**			
Colombiana	10	9	0,686 ^||^
Venezolana	4	5	
**Estado civil**			
Soltera	4	0	0,094 ^||^
Casada	1	1	
Unión libre	9	13	
**Primer hijo**			
Si	7	9	0,445 ^||^
No	7	5	
**Parto múltiple**			
Si	2	3	0,622 ^||^
No	12	11	
**Peso para la edadgestacional**			
Bajo peso	4	2	0,648 ^||^
Peso adecuado	10	12	
**Cuenta con el apoyodelpadre**			
Si	11	14	0,067 ^||^
No	3	0	

*n = Muestra; ^†^Sig = Significancia estadística; ^‡^t = Prueba t de Student; ^§^U = Prueba U de Mann-Whitney; ^||^Chi^2^ = Prueba Chi^2^

En cuanto al efecto preliminar, en la comparación entre grupos, mediante la aplicación de una t de Student para muestras independientes, se pudo establecer que en ambos mejoró la competencia para el cuidado, sin embargo, la diferencia no fue significativa. Medición basal: Control (n=14) 81,43 ± 15,37 vs Intervención (n=14) 80,71 ± 16,85 p=0,908; medición al alta: Control (n=13) 117,08 ± 6,103 vs Intervención (n=14) 121,14 ± 7,156 p=0,126 y medición una semana después del alta: Control (n=7) 112,56 ± 6,241 vs Intervención (n=9) 118,67 ± 9,421 p=0,163.

Por su parte en la comparación intragrupo, mediante la prueba t de Student para muestras relacionadas, se identificó que el nivel de competencia al momentos del alta hospitalaria aumentó respecto a la medición basal, encontrando una diferencia estadísticamente significativa a favor de la intervención, por lo que se calculó la “d” de Cohen para determinar el tamaño del efecto: Control (n=13): basal 82,15 ± 15,74 vs al alta 117,08 ± 6,10 p=0,000, d de Cohen 12,72; Intervención (n=14) basal 80,71 ± 16,85 vs al alta 121,14 ± 7,15 p=0,000, d 18,55.

Esta misma tendencia se identificó en la medición una semana después del alta respecto a la medición basal: Control (n=7): basal 86,71 ± 17,14 vs una semana después del alta 112,57 ± 6,24 p=0,004, d 15,302; Intervención (n=9) basal 81,22 ± 16,10 vs una semana después del alta 118,67 ± 9,421 p=0,000, d 16,853.

Para el caso de la diferencia entre el momento del alta y una semana después se encontró: Control (n=7) alta 117,43 ± 7,829 y una semana después del alta 112,57 ± 6,241 p=0,192 vs Intervención (n=9) alta 121,33 ± 7,365 y una semana después del alta 121,14 ± 7,156 p=0,335; pero la diferencia no fue estadísticamente significativa.

En cuanto al efecto de la intervención ACUNE sobre variables secundarias de importancia, solo se encontraron diferencias estadísticamente significativas entre los grupos de estudio, en los días de estancia en cuidados básicos: Control n=13: 11,38 ± 10,054 vs Intervención n=13: 4,14 ± 2,656 p=0,025).

Se exploró la posible interacción de las variables que pudieran intervenir en la diferencia de las puntuaciones. Para tal efecto se aplicó una ANOVA de medidas repetidas en función de las tres mediciones de la competencia del cuidador y no se encontraron diferencias estadísticamente significativas que indicaran que alguna de ellas pudiera influir en el resultado de la competencia para el cuidado.

Por último, se identificó que la intervención ACUNE tiene una adecuada aceptabilidad. Todas las madres indicaron que la intervención les pareció importante, útil, clara y pertinente. Con relación a la estructura de la intervención se encontró que el 50% de las madres percibió que el tiempo de cada sesión fue corto y el 57.1% que las sesiones eran pocas. Dentro de la evaluación de la aceptabilidad también se indagó acerca de la cartilla, su utilidad, pertinencia, presentación y claridad, fue valorada entre “util” y “muy util” por todas las madres. Su presentación, claridad y contenido fueron estimados como excelentes, por la mayoria de lasmadres.

En la Figura 4 se presenta la conexión de los datos mediante exhibición conjunta, en donde los hallazgos cualitativos buscan explicar y complementar los cuantitativos estadisticamente significativos obtenidos en la segunda fase, para generar las metainferencias de los datos.

**Figura 4-:** Exhibición conjunta de datos y metainferencias

**Resultados cuantitativos** **Competencia para el cuidado (Intragrupo)**	**Resultados cualitativos** **Empoderandose para el cuidado: testimonios**	**Metainferencias**
**Grupo Control (n=13)** Medición basal 82,15 ±15,74 Al alta 117,08 ± 6,10 p=0,000 **Grupo Intervención (n=14)** Medición basal 80,71 ±16,85 Al alta 121,14± 7,15 p=0,000	*[…] Yo creo que uno se tiene que empoderar, estos son mis hijos y esto es lo que me va a tocar hacer con ellos y lo tengo que aprender sin miedo […]* (P5) *.* *[…] Echar para adelante porque para atrás no podemos, es empoderarnos y aceptar la situación como venga, cuando uno entiende eso es cuando aprende, algunas enfermeras me decian que yo podia, que confiara en mi, que si otras mamás podian, yo tambíen podía y eso fue lo que más me ayudó* *[…]* (P11) *.*	La intervención cuyo enfoque central fue el empoderamiento demostró de forma preliminar, que mejora la compentencia para el cuidado, guardando coherencia con las experiencias de los padres, quienes informaron que empoderarse fue esencial para aprender a cuidar a sus hijos. Los padres necesitan más que conocimientos y es necesario apoyarlos para vencer los miedos ante la fragilidad de sus hijos y para vincularse con el cuidado dentro de la unidad neonatal. Una intervención de enfermería que empodera presenta un efecto positivo sobre la compentencia para el cuidado.
**Grupo Control (n=7)** Medicion basal 86,71 ±17,14 Una semana después del alta 112,57 ± 6,24 p=0,004 **Grupo Intervención (n=9)** Basal 81,22 ± 16,10 Una semana después del alta 118,67 ± 9,421 p=0,000	*[…] Yo decía si me la van a dejar llevar para la casa es porque ella está bien y ven que ella puede respirar solita y porque yo voy a poder cuidarla […* (P8). *[…] Finalmente va a ocurrir lo que estábamos esperando, estar preparados para estar en nuestro hogar […]* (P5) *.* *[…] ya veníamos para la casa, en cambio allá estábamos con las enfermeras y no era sino tocar un botón y solucionábamos el problema, acá en la casa si le da a uno mucho susto […]* (P9).	La intervención fue diseñada para promover el empoderamiento después del alta hospitalaria y los resultados del estudio experimental mostraron un efecto a favor de la intervención en este sentido. Si bien los resultados son preliminares, orientan hacia la conveniencia de incluir este enfoque teórico en los procesos de preparación para el alta. Por otra parte, en los resultados se puede identificar que el nivel de competencia al alta es mayor que el nivel registrado una semana después del alta, lo cual indica que la primera semana en el hogar es crítica para los padres, quienes enfrentan la realidad del cuidado independiente, aspecto que fue informado en las entrevistas.
**Días de estancia en cuidados básicos** Control (n=13): 11,38 ± 10,054 Intervención (n=13): 4,14 ± 2,656 p=0,025 En la aceptabilidad, el 50% de las madres percibió que el tiempo de cada sesión fue corto y el 57.1% que las sesiones eran pocas	*[…] Yo ya me quería venir para la casa, yo ya me sentía preparada, yo ya lo había hecho, lo hice allá en la unidad, yo ya queria salir rápido, pero porque ya sabía como cuidar a mi hijo y me sentía segura […]* (P5). *[…] Yo todo decía, ¡yo lo hago! Y me preguntaban ¿Usted si es capaz? Les decia no, pero yo tengo que aprender, ¿entonces cuando me lo lleve que voy a hacer? no las voy a tener, yo quiero aprender acá, aprender de ustedes y en cuanto mas me enseñen mucho mejor […]* (P1).	De forma preliminar los resultados muestran que la intervención disminuye los días de estancia en cuidados básicos, que es el contexto de cuidado previo al alta. Las madres al serntirse empoderadas se sienten seguras y demuestran su compentencia al momento de valorar este criterio de alta. Considerado el empoderamiento como elemento diferencial, además del material y la forma de entrega, las madres esperan contar con mas sesiones y tiempo bajo esta perspectiva, desde sus experiencia un mayor acompañamiento directo genera mas confianza para el cuidado.

## Discusión

Los hallazgos cualitativos ratifican la complejidad y multidimensionalidad de tener un hijo prematuro^(3)^. La preparación para el alta no es un proceso aislado, se conjuga dentro de las vivencias en torno al nacimiento, la hospitalización en la unidad neonatal y el cuidado del hijo en el hogar. Los participantes no definen un inicio y un final en términos de la preparación para el cuidado de sus hijos; reafirmandolo como un proceso continuo que comienza desde el ingreso a la unidad neonatal y no termina con el alta hospitalaria^(3,23)^.

La experiencia de los participantes inicia con el nacimiento prematuro y con las vivencias que los confrontan con una realidad inesperada. Posteriormente, se familiarizan con la cotidianidad de la unidad neonatal donde se vinculan con su hijo, se empoderan y preparan para el cuidado en el hogar. Por último, llegan a su hogar y se enfrentan a una realidad donde el cuidado, el apoyo y los programas de seguimiento cumplen un papel fundamental en la experiencia. Estos hallazgos, son consistentes con otros estudios de tipo cualitativo, en los que se han explorado expriencias de padres de niños prematuros, encontrando que al igual que en este, la hospitalización y la transición al hogar es una trayectoria compleja y dinámica hacia el cuidado competente en el hogar^(23-25)^. Estos hallazgos fueron integrados en el diseño de la intervención, en su aplicación y en los procedimientos mismos del estudio experimental; encontrando a través de la integración de los métodos mixtos, que los resultados son consistentes y se cofirman. Al igual que en las experiencias de los padres en donde expresaron que cuando el personal de enfermería les ayudaba a sentirse capaces y empoderados lograban cuidar mejor a sus hijos, la intervención intencionada y orientada hacia el empoderamiento, logró un efecto preliminar positivo a favor de la intervención.

La cotidianidad de la unidad neonatal está marcada por necesidad de información acerca de la salud de los hijos y de las dinámicas de atención propias del contexto, factores ya identificados en otros estudios^(22-24)^. Esto orienta hacia la pertinencia de incluir información para la familiarización con la unidad neonatal, pues es desconocida y estresante^(24-26)^. Este aspecto fue fundamental en la intervención y fue el eje central de la primera sesión, generando una perspectiva de pertinencia en el abordaje inicial del proceso educativo.

Desde la experiencia de los participantes, el empoderamiento es central en el fortalecimiento de la competencia para el cuidado del hijo en el hogar, pues surge de asumir su rol parental y de sentirse responsables del cuidado de sus hijos. Estos eventos movilizan fuerza y determinación para involucrarse en el cuidado y vencer los temores. La información, el apoyo del personal de enfermería y de la familia aportan al empoderamiento, el cual inicia en la unidad neonatal y se fortalece al alta. La necesidad del empoderamiento ha estado presente en otros estudios, que reafirman que el apoyo en la participación activa del cuidado del hijo dentro de la unidad es un mediador del empoderamiento, que ayuda a la seguridad y confianza para la transición^(27-28)^. La intervención ACUNE, al centrarse en el empoderamiento, se ubica en la experiencia de los padres y las toma como punto de partida, para comprobar que este enfoque genera un resultado positivo sobre la experiencia y sobre la capacidad de cuidado de los padres.

El empoderamiento ha sido abordado en la educación para la salud como parte de la tendencia hacia una atención y un cuidado participativo^(29-30)^. Esto reafirma el sentido del empoderamiento dentro de los procesos de preparación de los padres para el alta de la unidad neonatal y favorece la integración de la teoría del empoderamiento a la intevención ACUNE.

De acuerdo con la teoría, el empoderamiento para la salud es un proceso relacional que surge del reconocimiento de la persona, de sus recursos personales y del contexto social^(19-20)^. Este principio guarda coherencia con las vivencias de los participantes y se constituye como aspecto central de la intervención ACUNE, cuya evaluación preliminar indica que es viable, aceptable y que puede generar un efecto positivo sobre la competencia para el cuidado del hijo prematuro. La intervención demostró ser viable en función de los recursos físicos y humanos. La simpleza de su estructura hace posible que pueda ser entregada a las madres de acuerdo con lo planeado. Durante todo el estudio se encontró viable la medición de las variables de interés y los instrumentos fueron aplicados sin dificultades.

Al analizar los resultados de la competencia para el cuidado en el hogar entre grupos, no se encontraron diferencias estadísticamente significativas, pero se identificó que la puntuación de la competencia aumentó en ambos grupos, siendo mayor en el grupo de intervención. Las madres de ambos grupos recibieron la intervención habitual, pero las madres del grupo de intervención recibieron de forma adicional la intervención ACUNE que, a diferencia de la intervención habitual, tiene una estructura definida y articulada a un marco teórico disciplinar deempoderamiento.

Al evaluar el efecto intragrupo, se pudo evidenciar que existen cambios favorables en ambos grupos según momentos de medición y en este análisis se identificaron diferencias estadísticamente significativas. Este resultado preliminar indica que la intervención podría mejorar la competencia de las madres para el cuidado de sus hijos prematuros en el hogar, lo que sugiere que resulta pertinente realizar el estudio aleatorizado a mayor escala.

Al comparar los resultados con otros estudios que han buscado establecer el efecto del enfoque de empoderamiento, es importante reconocer que la evidencia da cuenta que tiene efectos favorables. Un reciente estudio realizado con 80 madres, 40 en cada grupo, encontró que una intervención de consejería orientada a las madres de niños prematuros, tuvo una diferencia significativa en la puntuación media de autoestima 9,52 ± 3,22 y 16,75 ± 6,39 (p<0,001) y de estrés 154,65 ± 32,15 y 61 ± 10,98 (p<0,001). Dentro del estrés, la reducción más importante se identificó en la dimensión de apariencia y comportamiento del lactante (d de Cohen=3,146)^(29)^. Este hallazgo reafirma la coherencia de la intervención ACUNE con la evidencia, pues se incluyó información relacionada con este tema, por los hallazgos de la primera fase, en donde a través de la experiencia de los padres, se identificó que la apariencia inicial del hijo prematuro era impactante y generaba temor durante el cuidado y contacto directo con su hijo.

Un aspecto llamativo es que, si bien la medicion al alta y una semana después del alta en el grupo intervención es mayor respecto al grupo control, se identificó que hay una dismución en la puntuación despues del alta, lo cual tiene que ver con que la primera semana de la transicion al hogar es crítica, y los padres enfrentan nuevos retos y desafios que pueden cuestionar su capacidad y su confianza puede verse disminuida^(28)^.

En el presente análisis, es necesario considerar que los resultados en relación con el efecto son preliminares. Al tener un tamaño de muestra pequeño, tiene poco poder estadístico, a lo que se suma para el caso particular el alto número de pérdidas. Estos aspectos, de algún modo pueden esperarse en los estudios piloto, razón por la cual las posibilidades de encontrar diferencias y de realizar otro tipo de análisis es limitada. Por lo tanto, la interpretación de los resultados es cuidadosa y no concluyente frente al efecto, aunque si orientadora; además, permite conocer aspectos relacionados con la viabilidad y aceptabilidad, optimizando tiempo y recursos al momento de realizar el ensayo clinico aleatorizado.

La aceptabilidad de la intervención fue buena y el principal ajuste sugerido después del piloto, tiene que ver con la viabilidad del seguimiento a las madres que son remitidas a otras instituciones. Al respecto, es necesario asegurar y clarificar el acceso a las madres para realizar la medición después del alta, utilizando medios virtuales para disminuir las pérdidas.

Estos resultados reafirman que el empoderamiento para la salud, es una perspectiva teórica que permite mejorar las capacidades y la confianza de las madres para el cuidado de sus hijos prematuros. Surge desde la experiencia de los padres y se confirma al evaluar una intervención orientada por este enfoque, que resulta socialmente sensible, validada en las experiencias de los padres y confirmada de forma preliminar mediante un estudio experimental que abre posibilidades a nuevas fases para la confirmación del efecto y que le da valor y sentido a la investigación de métodos mixtos, que envuelve las voces de las personas involucradas en los fenómenos de interés para la disciplina.

La evaluación preliminar de la intervención ACUNE, indica que podría generar un efecto positivo en la competencia para el cuidado de un hijo prematuro en el hogar. Los resultados tienen carácter preliminar, no son concluyentes, pero si orientadores frente a la conveniencia de la realización del estudio principal.

Se considera como limitación el tamaño de la muestra del estudio piloto. Si bien para este tipo de estudio no se genera mediante cálculos formales y el alcance es orientador, una muestra de mayor tamaño podría tener una representación mas cercana del efecto real. Sin embargo, se reconoce desde su perspectiva mixta como fortaleza, el diseño de un intervención que surge desde las voces de los padres y la confirmación preliminar del empoderamiento como un enfoque teórico que mejora la compentencia para el cuidado de los hijos prematuros.

## Conclusión

La experiencia de madres y padres en el desarrollo y fortalecimiento de la competencia para el cuidado del hijo prematuro es compleja y dinámica. El empoderamiento es un aspecto central, que favorece la capacidad de cuidado, la confianza y la transición al hogar. La participación en el cuidado, el apoyo de la familia y el acompañamiento y la interacción con el personal de enfermería son esenciales para el empoderamiento. El diseño de una intervención de enfermería para fortalecer la competencia para el cuidado de los hijos prematuros desde el enfoque del empoderamiento tiene una base teórica que permite su aplicación en la práctica; además, existe evidencia empírica que soporta la conveniencia de este tipo de intervenciones. La integración de los resultados cualitativos al diseño de la intervención favoreció su sensibilidad social y cercania con la realidad de las madres y con la práctica profesional de enfermería.
